# Osmoregulatory evolution of gills promoted salinity adaptation following the sea–land transition of crustaceans

**DOI:** 10.1007/s42995-025-00298-6

**Published:** 2025-05-15

**Authors:** Hongguang Liu, Xiaokun Wang, Zeyu Liu, Shuqiang Li, Zhonge Hou

**Affiliations:** 1 State Key Laboratory of Animal Biodiversity Conservation and Integrated Pest Management, Institute of Zoology, Chinese Academy of Sciences, Beijing, 100101 China; 2 School of Life Sciences, Hebei University, Baoding, 071002 China; 3 University of Chinese Academy of Sciences, Beijing, 100049 China

**Keywords:** Genetic adaptation, Gill, Habitat transition, Ion transport

## Abstract

The sea–land transition is one of the most dramatic evolutionary changes and requires an adaptive genetic response to salinity changes and osmotic stress. Here, we used multi-species genomes and multi-tissue transcriptomes of the talitrid crustaceans, a living sea–land transition model, to investigate the adaptive genetic changes and osmoregulatory organs that facilitated their salinity adaptation. Genomic analyses detected numerous osmoregulatory genes in terrestrial talitrids undergoing gene family expansions and positive selection. Gene expression comparisons among species and tissues confirmed the gill being the primary organ responsible for ion transport and identified the genetic expression variation that enable talitrids to adapt to marine and land habitats. V-type H^+^-ATPases related to H^+^ transport play a crucial role in land adaptations, while genes related to the transport of inorganic ions (Na^+^, K^+^, Cl^−^) are upregulated in marine habitats. Our results demonstrate that talitrids have divergent genetic responses to salinity change that led to the uptake or excretion of ions in the gills and promoted habitat adaptation. These findings suggest that detecting gene expression changes in talitrids presents promising potential as a biomarker for salinity monitoring.

**Supplementary Information:**

The online version contains supplementary material available at 10.1007/s42995-025-00298-6.

## Introduction

Salinity is a crucial factor for successful transition to land of marine-derived crustaceans (McNamara and Faria 2012; Stern and Lee 2020). Organisms must evolve numerous genetic changes, such as gene family evolution and RNA expression variation, to maintain their internal salt concentrations and ion homeostasis through active transport of ions against concentration gradients across permeable membranes (Cui et al. 2021; Liu et al. 2023; Yuan et al. 2021). To explore the adaptive response and genetic regulatory patterns to salinity changes, a continuous sea–land transition model would be desirable.

Terrestrial talitrid crustaceans inhabit humid areas near freshwater lakes or streams, while coastal talitrids live in intertidal zones affected by daily tidal changes. Previous studies revealed that the geological events of sea–land transitions promoted the divergence between terrestrial and coastal species (Liu et al. 2023). For instance, *Morinoia aosen,* a terrestrial talitrid belonging to the *Morinoia japonica* species complex from East Asia, was recently shown to have diverged with the Northwest Pacific marine coastal talitrid *Platorchestia pacifica* species complex via a Miocene marine incursion (Liu et al. 2022; Yang et al. 2013)*.* Although the genus *Platorchestia* is not monophyletic and contains some terrestrial species (e.g., *Platorchestia hallaensis*), *M. aosen* and *P. pacifica* have long been treated as a sea–land pair (Yang et al. 2013). The habitat salinity difference between this sea–land pair (*M.aosen* habitat salinity 0.48–0.51 ppt and *P. pacifica* habitat salinity: 4.8–37.5 ppt) led to a significant difference in their hemolymph osmolality (*M. aosen* 943–978 mOsm/kg and *P. pacifica* 1228–1340 mOsm/kg). This indicates that osmoregulation of salinity concentration should be the key to a successful habitat transition (Brooks and Millls 2006). However, the vital organ and genetic mechanisms related to osmoregulation and salinity adaptation following land colonization remains unclear.

The crustacean gill has been hypothesized to be a multi-functional organ that participates in several physiological processes, including ion transport and respiration (Friend and Richardson 1986; Henry et al. 2012; Tsai and Lin 2007). Preliminary work suggests that terrestrial talitrids use their gills for ion uptake, as do their aquatic relatives, and osmoregulatory sites appear to be distributed throughout the gill (Spicer et al. 1987; Swain and Richardson 1993). Despite this, little evidence from tissue comparison supports the assertion that gills play a major role in ion transport and terrestrial survival. Therefore, further genetic comparison and functional investigation between gills and other tissues would shed light on their osmoregulatory mechanisms and their role in adapting to a terrestrial lifestyle.

Comparative transcriptomic analyses have shown that marine and freshwater crustaceans exhibit different genetic mechanisms for salinity change according to the utilization of several enzymes that are involved in regulating the flow of inorganic ions (such as Na^+^, H^+^, and Cl^−^) into and out of cells to maintain hemolytic osmotic balance (Yuan et al. 2017). For example, Na^+^/K^+^-ATPase (Na/K-ATPase) is the driving force in establishing an ion gradient across the epithelial cell membrane in marine crabs, whereas freshwater crabs may use V-type H^+^-ATPases by generating the H^+^ ion gradient to facilitate ion flow (McNamara and Faria 2012). A comparative analysis based on sea–land species pairs can detect fine-scale genetic variation to allow the quantification of specific environmental stress adaptation mechanisms.

In this study, to investigate the osmoregulatory mechanism following land colonization, we first used comparative genomic methods to study adaptive genetic changes and variation based on seven amphipod species genomes. Then, we investigated the expression patterns between five tissues from a sea–land pair (*M. aosen* and *P. pacifica*) to identify the organs involved in osmoregulation. We also compared the variation of genetic expression of gills between *M. aosen* and *P. pacifica* to uncover the potential role of the gills in osmoregulation and other adaptive mechanisms in different habitats. Lastly, we used gradient salinity stress experiments to verify the detected gene expression changes and summarized the genetic regulatory mechanisms under salinity changes. Our results will contribute to the understanding osmoregulatory mechanism of gills following the sea–land transition, which provides an important basis for further research on genetic changes during the adaptation to land by organisms that originated in the sea.

## Materials and methods

### Species selection

To perform genomic comparison analyses, we first constructed an evolutionary framework based on the following criteria: (1) at least two species within each habitat; (2) a close phylogenetic relationship consistent with sea–land transition. Based on the first criterion, we downloaded genome sequences and protein-coding genes of two terrestrial talitrids, *M. aosen* (GCA_030386875.1) and *P. hallaensis* (GCA_014220935.1), and two coastal species, *Trinorchestia longiramus* (GCA_006783055.1) and *Orchestia grillus* (GCA_014899125.1), from the National Center for Biotechnology Information (NCBI). The genus *Platorchestia* is not monophyletic and needs revision because it contains both terrestrial (e.g., *P. hallaensis*) and coastal species (e.g., *P. pacifica*). The terrestrial species show a closer relationship with *Morinoia aosen*. Thus, we also included the genome of the coastal species *P. pacifica* in the analyses to satisfy the second criterion. Two aquatic amphipods (*Hyalella azteca* and *Parhyale hawaiensis*, which are euryhaline or adapted to seawater) were selected as outgroups (Supplementary Table S1).

### Genome update of *Platorchestia pacifica*

To better study the genome evolution of talitrids following the sea–land transition process, we reassembled and reannotated the genome of *P. pacifica*. Cleaned sequencing reads were assembled using MEGAHIT v1.2.9 (Li et al. 2015). The contig-level assembly was scaffolded to a pseudochromosome scale with RagTag v2.0.0 (Alonge et al. 2022) using *M. aosen* (GCA_030386875.1) as a reference. Three strategies were applied for gene structure prediction across the genome: homology-based annotation, de novo prediction, and RNAseq-based annotation. For homology-based annotation, the published genomes of three amphipods, *H. azteca*, *T. longiramus*, and *M. aosen*, were used. We independently ran the module GeMoMa pipeline for each species using MMseqs2 (Steinegger and Söding 2017) and combined the three gene annotations using the GeMoMa modules GAF and AnnotationFinalizer. The de novo gene prediction and transcriptome-based prediction were performed following Liu et al. 2023.

### Measurement of salinity and osmolality

We focused on a sea–land pair, *P. pacifica* and *M. aosen,* to determine the osmolality of the environment and hemolymph. We first measured the salinity of each habitat with a water quality meter (AZ 86031). The *P. pacifica* specimens were collected from Beidaihe, Hebei province, China, where their environmental salinity was 4.8–37.5 ppt. During the field collection, we also collected the seawater and sand which was transferred with talitrids into a vivarium thus ensuring minimal salinity difference during tissue dissection and RNA extraction in the laboratory. The *M. aosen* specimens were collected along a freshwater lake in Olympic Forest Park, Beijing, China, and then kept in a vivarium with water from the lake. The environmental salinity of the collection site was 0.48–0.51 ppt. The hemolymph of *M. aosen* and* P. pacifica* was collected from 10 to 15 individuals using a microcapillary tube to extract fluid from between the second and third abdominal segments. The osmolality of each habitat and of the hemolymph of *M. aosen* and* P. pacifica* was measured with a Fiske 210 Micro-Sample Osmometer (Advanced Instruments)*.* All data were obtained from three replicates.

### Comparative genomics and gene family analysis

To detect the adaptive evolution of amphipods following the sea–land transition, we performed gene family analysis and positive selection analysis based on the genome sequence of coastal and terrestrial talitrids. OrthoFinder v2.5.4 was used to define gene families based on protein-coding genes (Emms and Kelly 2019). The species tree generated from OrthoFinder was used in the following analysis. The amphipods’ divergence time was estimated using the r8s v1.81 (Sanderson 2003) based on the divergence time of *T. longiramus* and *Parhyale hawaiensis* (132 million years ago) according to Timetree (http://timetree.org/) and Cannizzaro et al. (2022).

Gene family expansion and contraction analyses were performed using CAFE v4.2 (De Bie et al. 2006).The positively selected genes (PSGs) were identified using the branch-site model and likelihood ratio test based on the one-to-one orthologs, which were obtained using the reciprocal best hit (RBH) method (Moreno-Hagelsieb and Latimer 2008) with *M. aosen* as a reference species. Two terrestrial species (*M. aosen* and *P. hallaensis*) were set as foreground branches in PAML v4.9j (Yang 2007). The null model (model = 2, NSsites = 2, fix_omega = 1) and an alternative model (model = 2, NSsites = 2, fix_omega = 0) were used, and *P*-values were calculated using a chi-square distribution with one degree of freedom. Genes with a dN/dS value larger than 1 and false discovery rate (FDR) less than 0.05 were identified to be under positive selection. Functional enrichment analyses of the expanded gene family and PSGs were performed with KOBAS 3.0 (Bu et al. 2021) using Fisher’s exact test (*P* < 0.05) and *Drosophila melanogaster* as a reference.

### Specifically expressed gene identification for each tissue

For the gene expression analyses, we extracted RNA of *M. aosen* and *P. pacifica* from gills, compound eyes, nerves, antennae, and pereopods with a mixture of tissues from 10 to 15 individuals using the RNAsimple Total RNA kit (TIANGEN, Beijing, China). The transcriptomes sequencing strategies were same as a parallel study (Liu et al. 2023), and gills, pereopods, and compound eyes were newly obtained in this study. Based on the aligned and consistent length orthologs between these two species, the clean RNA-seq reads of five tissues were mapped to their own trimmed orthologs. For each species, the expression level (fragments per kilobase million and expected count) of orthologs was calculated with RSEM v1.3.3 (Li and Dewey 2011) and log-transformed (N + 1) following the quantile-normalization in the R package limma v3.40.6 (Ritchie et al. 2015). Ortholog gene expression matrices of *M. aosen* and *P. pacifica* were obtained after removing the batch effect with the R package sva v3.32.1 (Leek et al. 2012).

To identify the potential function of each tissue, we identified specifically expressed genes by calculating tissue specificity indices *τ* (Kryuchkova-Mostacci and Robinson-Rechavi 2017). *τ* is defined as follows:

$$\tau = \frac{{\mathop \sum \nolimits_{i = 1}^{n} \left( {1 - \widehat{{X_{i} }}} \right)}}{n - 1};\;\widehat{{X_{i} }} = \frac{{X_{i} }}{{\mathop {\max }\limits_{1 \le i \le n} X_{i} }}$$

*X*_*i*_ is the expression of the gene in tissue *i*, and *n* is the total number of tissues. The *τ* value ranges from 0 to 1: a value close to 1 indicates tissue-specific expression, while a value close to 0 indicates ubiquitous expression. Specifically expressed genes were defined as those that had *τ* values > 0.9 and the samples with the highest expressions were obtained from the same tissue type. We analyzed the specifically expressed genes in *M. aosen* and *P. pacifica* separately, and the genes in the gills of both species were treated as common genes.

### Semantic similarity permutation between two sets of genes

To evaluate the overall similarity of the functional enrichment patterns between two sets of genes, we calculated pairwise semantic similarity values of the Gene Ontology (GO) terms and examined semantic similarity (SS) values relative to random samples with the GoSemSim package in R based on BMA algorithms (Yu et al. 2010). To determine if the observed semantic similarity values deviated notably from random expectation, we designed a simulation test by sampling the same numbers of GO terms from the GO categories of *Drosophila melanogaster* 1000 times, calculating the SS values each time. For permutations, observed SS values greater than or equal to the 95th percentiles of the random samples were considered significant.

### RNA expression patterns and differential expression analyses between the sea–land pair

For transcriptome expression similarity comparisons, principal component analysis (PCA) was conducted using the “plotPCA” function in the DESeq2 package v1.24.0 based on the ortholog gene expression matrices (Love et al. 2014). Hierarchal-clustering expression profile analysis was conducted based on Spearman’s correlation coefficients of gene expression between pairs of samples.

To classify gene association patterns in different tissues, the co-expression networks of five tissues from *M. aosen* and *P. pacifica* were constructed using the weighted gene co-expression network analysis (WGCNA) (Langfelder and Horvath 2008). A height cutoff of 0.2 and a minimum module size of 100 were applied to merge similar expression profiles with nine modules obtained. The hub genes in the co-expression network were identified based on module membership correlations (more than 0.8) and significances between gene and trait (absolute value > 0.2). Network visualization for each module was performed using Cytoscape v3.6.1 with a cut-off of the weight parameter obtained from the WGCNA set at 0.3 (Jeong et al. 2001).

Differentially expressed genes (DEGs) of tissues and species were identified using R package DESeq2. The threshold value of DEGs was set to *P*-value < 0.05 and absolute of fold change ≥ 2 (log_2_|FC|≥ 1). Three comparisons were conducted: (1) DEGs of gills in *M. aosen* and *P. pacifica*; (2) DEGs of gills relative to other tissues in *M. aosen*; (3) DEGs of gills relative to other tissues in *P. pacifica*. For the second and third comparisons, we first calculated the DEGs of the gills to other four tissues (antennae, compound eyes, nerve, and pereopods), and the genes that are common to all four sets were retained as DEGs of gills.

To identify whether some gene sets were significantly differentially expressed in gills between the two species, we performed gene set enrichment analysis (GSEA) to analyze their specific expression in both species using the clusterProfiler package in R (Wu et al. 2021). We focused on the GO pathways that have been shown to be related to osmotic pressure regulation. We also performed gene set variation analysis (GSVA) using the GSVA package (Hänzelmann et al. 2013). The gene expression matrices of *M. aosen* and *P. pacifica* and the gene list of GO terms were used as input data.

### Stress experiment under a salinity gradient

To validate the changes in RNA expression in response to the sea–land transition, adult talitrids of *M. aosen* were cultured in seawater using a salinity gradient (0.48 ppt, 5 ppt, 10 ppt, 20 ppt, and 35 ppt). Gills of *M. aosen* were collected individually for RNA extraction, and cDNA was generated using the HiScript cDNA Synthesis Kit (Vazyme) at 24, 48, and 72 h in each salinity treatment. The expression levels of 14 DEGs (*VATB*, *VATC*, *VATD*, *VATE*, *VATF*, *VATG*, *VATH*, *VATO*, *VATS*, *VAT116 KDa*, *SLC4 A5*, *SLC6 A13*, *SLC41 A1*, and *Chrna7*) were calculated using the 2^–ΔΔCt^ method. The *GAPDH* gene was used to normalize gene expression. All data were obtained from three independent assays with three replicates in each assay.

## Results

### Gene family expansion and adaptive evolution in amphipods

**Fig. 1:**
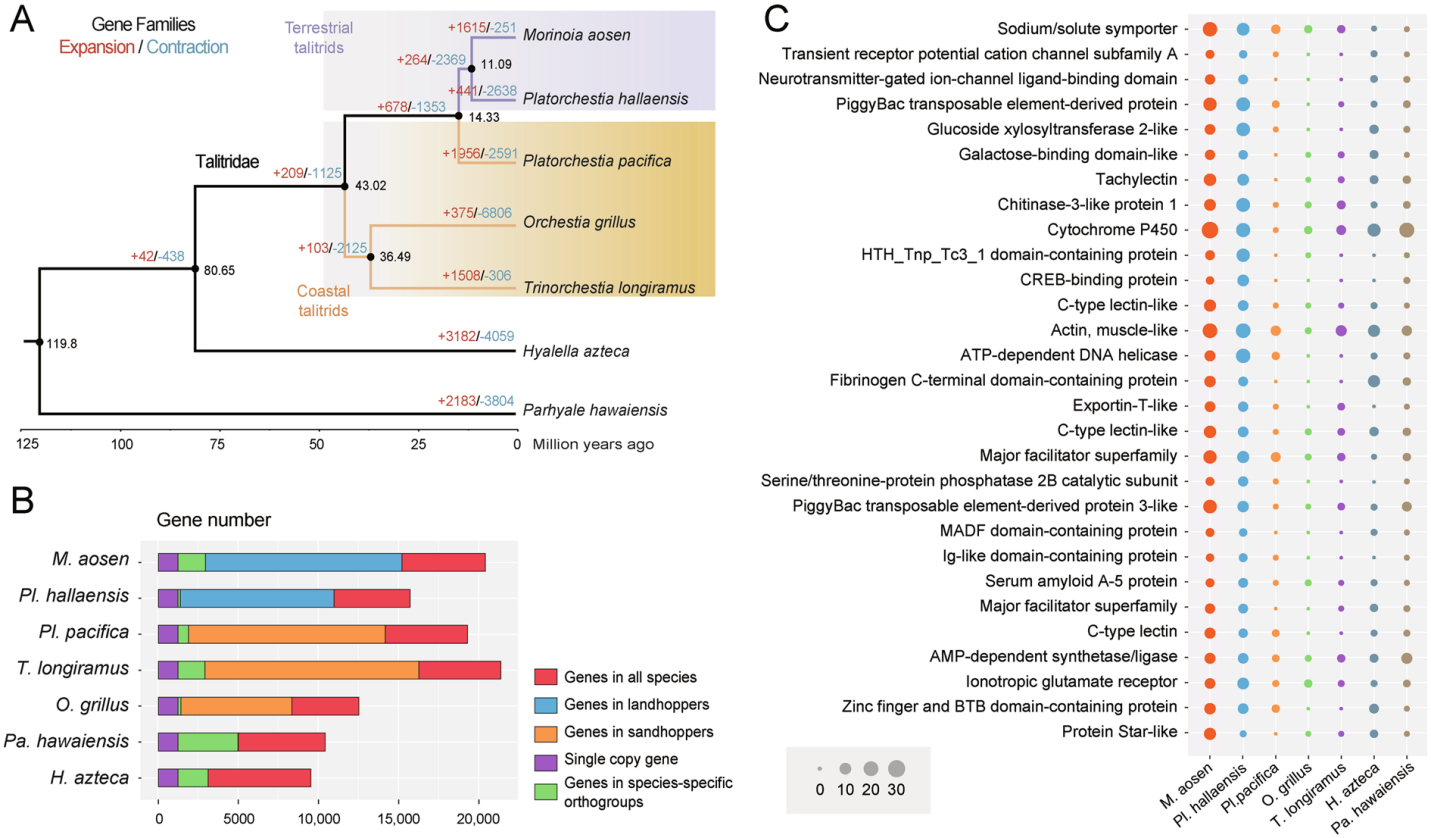
Comparative genomics analysis of seven amphipod species. **A** Gene family expansion/contraction analysis and phylogenetic tree of seven amphipod species. Expanded and contracted gene families are highlighted in red and blue, respectively. The estimated divergence times are displayed at each node. **B** Comparison of the gene repertoire of seven amphipod genomes. **C** Significantly expanded gene family in terrestrial talitrids. The size of the dots represents the number of genes in orthogroups

To better study the adaptive genetic changes following sea–land transitions, we reassembled and annotated the genome of *Platorchestia pacifica*. By using the BUSCO of arthropoda_odb10, we found the newly assembled genome obtained a higher completeness score of 89.4% than most published amphipod genomes. According to orthologous gene identification based on seven talitrid genomes, including two terrestrial species genomes (*Morinoia aosen* and *Platorchestia hallaensis*), three coastal species (*Platorchestia pacifica*, *Trinorchestia longiramus,* and *Orchestia grillus*), and two aquatic amphipods (*Hyalella azteca* and *Parhyale hawaiensis*) (Supplementary Table S1), a total of 17,646 families of homologous genes were detected in Amphipoda (Fig. 1A, B). A core set of 3632 gene families were shared by all species. Phylogenetic analysis based on 1226 single-copy orthologous genes suggests terrestrial species diverged from coastal species ~ 14.33 million years ago (Ma) (Fig. 1A).

As changes in gene copy number provide evidence of adaptive evolution, we examined the expansion of gene families between species from different habitats. The genomes of the terrestrial species *M. aosen,* and *P. hallaensis*, have 1772 and 146 species-specific genes, respectively (Fig. 1B). Based on the shared gene families, we found 29 gene families were significantly expanded in terrestrial species compared with coastal species, and these gene families were involved in transmembrane transport and ion channels according to the enrichment analysis (Fig. 1C; Supplementary Fig. S1).

We also identified 187 PSGs in the two terrestrial species as foreground branches and the two coastal species as background branches, based on one-to-one orthologs (Supplementary Table S2). Among these PSGs, we found some genes that encode transmembrane proteins or channels, including *TMC7* (Transmembrane channel-like protein 7), *TMC169*, *ORCT2* (Organic cation transporter-like protein 2), *EMC7* (endoplasmic reticulum membrane protein complex subunit 7), ABC transporter, and Epithelial sodium channel. Two PSGs encoding Ras GTPase (*RasGAP1*, *RasGAP2*) and one encoding Rab GTPase (*Rab3GAP2*), both of which belong to the family of GTPase, were also positively selected.

### Ortholog expression patterns of multiple tissues

To further detect the osmoregulatory organ and genetic variations related to osmoregulation following the sea–land transition, we compared the expression pattern between the tissue transcriptomes of a closely related sea–land pair, *M. aosen* and *P. pacifica*. We identified 9837 orthologous genes using the reciprocal best hit method based on the protein-coding genes from *P. pacifica* and the coding sequence of *M. aosen*.

Based on these orthologs, a total of 29 RNA-Seq datasets from five tissues (antennae, compound eyes, gills, nerve, and pereopods) were mapped to their own aligned and trimmed orthologs to obtain the ortholog gene expression matrix. Hierarchical clustering expression profile and principal component analysis suggest the gills and nerves split from the other three tissues and show a clear separation from each other, while the compound eyes, pereopods, and antennae cluster together (Supplementary Fig. S2).

**Fig. 2:**
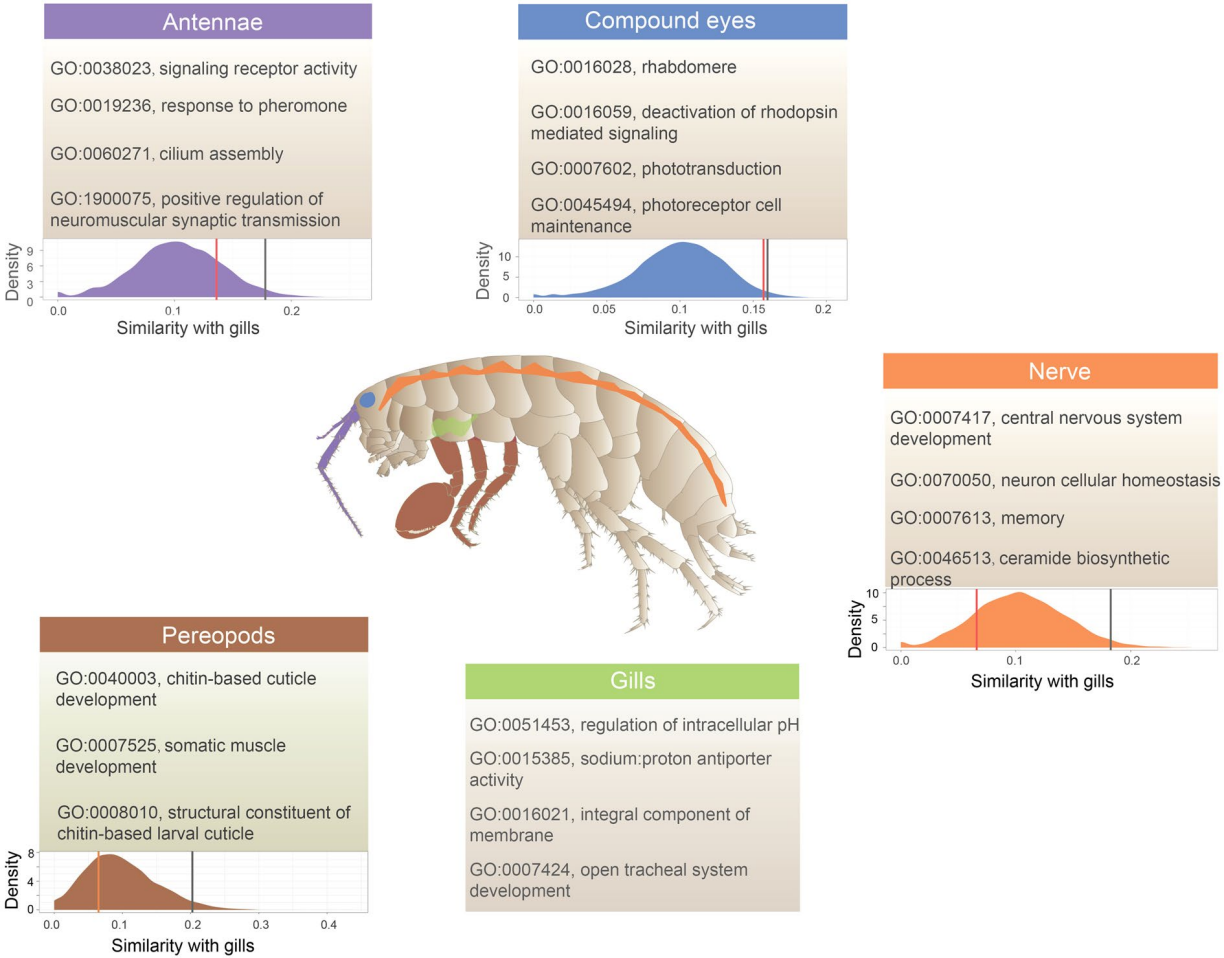
The enriched GO pathways of the specifically expressed genes that were identified in each tissue of *Morinoia aosen*. The density plots represent the observed pairwise semantic similarity (SS) scores and permutated scores between the gills and the other four tissues. The red vertical lines show the observed pairwise SS values. The filled area under the curve shows 1000 permutated SS values with 95th percentiles labeled as black vertical lines. Of these, the low similarity of the 95th percentile suggests that the gill does not show significant functional similarity with other tissues and is an important osmoregulatory organ

To further detect the detailed functional variation between tissues, we identified the specifically expressed gene in each tissue of *M. aosen* and *P. pacifica* based on the calculated tissue specificity indices τ (Kryuchkova-Mostacci et al. 2017). The genes identified in both species (τ > 0.9) were selected for GO functional enrichment analysis to give an idea of their potential function (Fig. 2). A total of 257 genes were specifically expressed in the compound eyes, 56 in the antennae, 514 in the nerves, 273 in pereopods, and 74 in the gills. According to the GO enrichment analysis (Supplementary Table S3), the significantly enriched pathways in each tissue were connected tightly with their function, e.g., rhabdomere (GO:0016028), and phototransduction (GO:0007602) in the compound eyes, signaling receptor activity (GO:0038023) and response to pheromone (GO:0019236) in the antennae, central nervous system development (GO:0007417) and memory (GO:0007613) in the nerve, and chitin-based cuticle development (GO:0040003) and somatic muscle development (GO:0007525) in the pereopods. Specifically, the enriched pathway in the gills shows their potential to function in osmoregulation and respiration simultaneously, e.g., regulation of intracellular pH (GO:0051453), sodium: proton antiporter activity (GO:0015385), and open tracheal system development (GO:0007424).

To further quantify the functional similarity or difference between the gills and other tissues, we calculated pairwise semantic similarity values of the GO terms and examined SS values relative to random samples. We randomly selected the same number of GO terms set 1000 times and calculated the SS values each time. Then, we compared the SS value between the two tissues to the distribution generated by the random selection. A higher SS value between two tissues than that of upper 95% confidence intervals would support the hypothesis that the two tissues have functional similarity, while a lower SS value would suggest the two tissues have functional differences. Our comparison results suggest that the GO terms of specifically expressed genes in the gills show no significant function similarity with the other tissues.

### Gill-related gene co-expression modules involved in osmoregulation

**Fig. 3:**
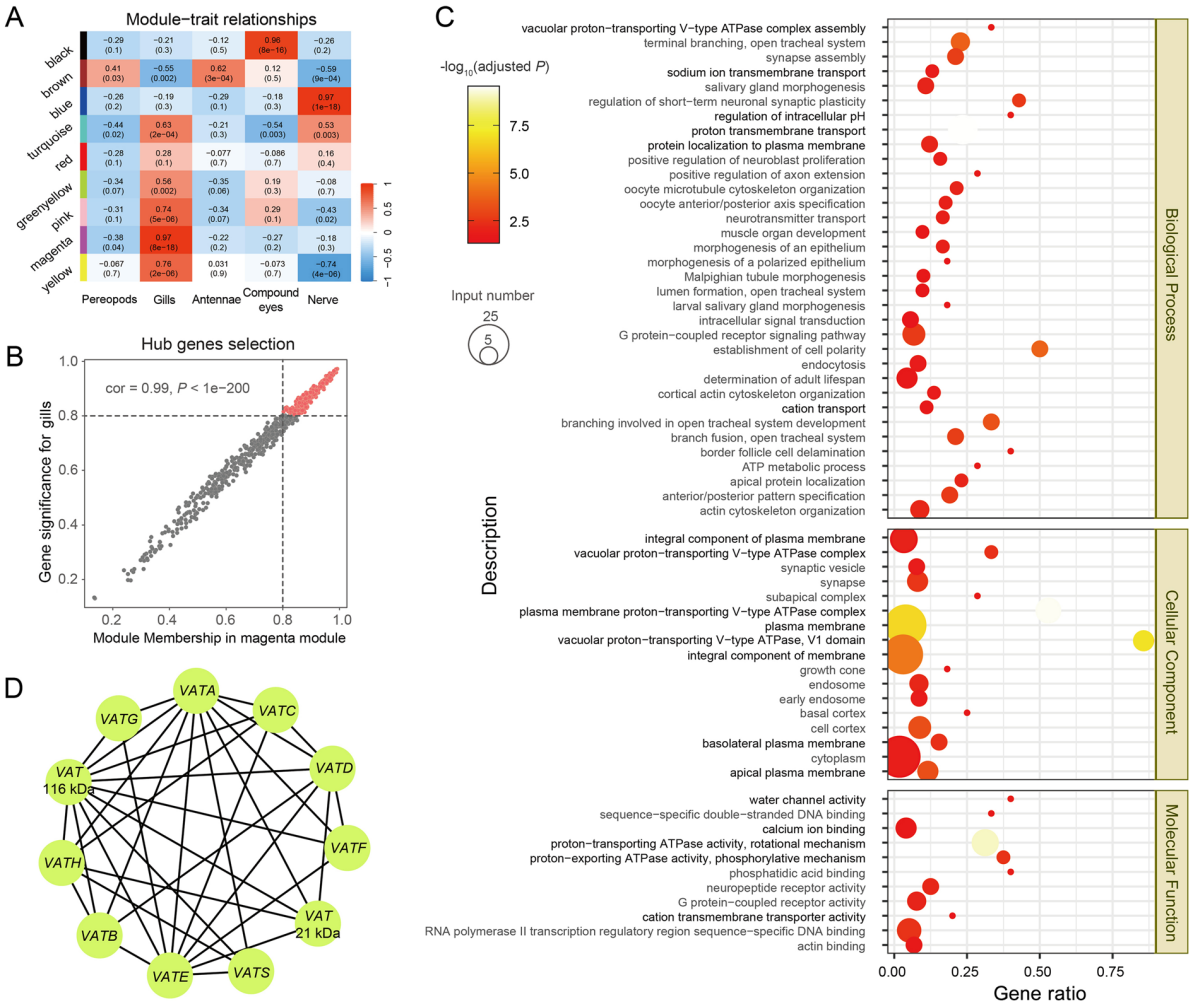
The gill-related module and core network related to osmoregulation. **A** Heatmap of correlation between modules and traits. Correlation coefficient along with *P*-value in parentheses below; color-coded according to correlation coefficient. **B** Scatterplot of gene significance (GS) for gills versus module membership (MM) in the magenta module. The genes with both GS and MM > 0.8 were selected as hub genes and labeled in red. **C** GO enrichment analysis of the hub genes. Circle size represents the number of annotations for certain GO terms, and the color indicates the corrected enrichment *P*-value on a log_10_ scale. GO terms related to osmoregulation are shown in black, and those that are not are shown in gray. **D** Cytoscape plot of V-type H^+^-ATPases genes with weight parameters > 0.3 in the core network module

To classify and identify the tissue-correlated modules, we performed co-expression network construction based on the gene expression matrix of *M. aosen* and *P. pacifica* with WGCNA. A total of nine co-expression modules were associated with different tissues based on 29 RNA-Seq datasets, each of which contained a different number of genes ranging in size from 171 to 2590 (Fig. 3A; Supplementary Fig. S3). Four co-expression modules were significantly correlated with gills, including magenta (*r* = 0.97, *P* = 8e − 18, 616 genes), yellow (*r* = 0.76, *P* = 2e − 6, 703 genes), pink (*r* = 0.74, *P* = 5e − 6, 377 genes) and turquoise (*r* = 0.63, *P* = 2e − 4, 2474 genes). The magenta module shows the highest module-trait associations with gills.

To acquire more detailed information about gene function in the magenta module, we checked and obtained 209 genes as the hub genes in the magenta module based on module membership correlations > 0.8 and significances between gene and trait (absolute value > 0.2; Fig. 3B). According to the pathway enrichment analysis for these hub genes in the magenta module, some crucial osmoregulation pathways were enriched, e.g., proton transmembrane transport (GO:1902600), sodium ion transmembrane transport (GO:0035725), vacuolar proton-transporting V-type ATPase complex (GO:0070072), and water channel activity (GO:0015250) (Fig. 3C).

To construct the co-expressed gene network based on the hub genes, we identified 19 genes with a weight parameter > 0.3 according to the node cytoscape results (Supplementary Table S4). Most of these (11/19) belong to the V-type H^+^-ATPases, which are large multi-subunit membrane-embedded complexes, including subunits A, B, C, D, E, F, G, H, S, 21 kDa, and 116 kDa (Fig. 3D).

### Differentially expressed genes between the sea–land pair are related to salinity changes

**Fig. 4:**
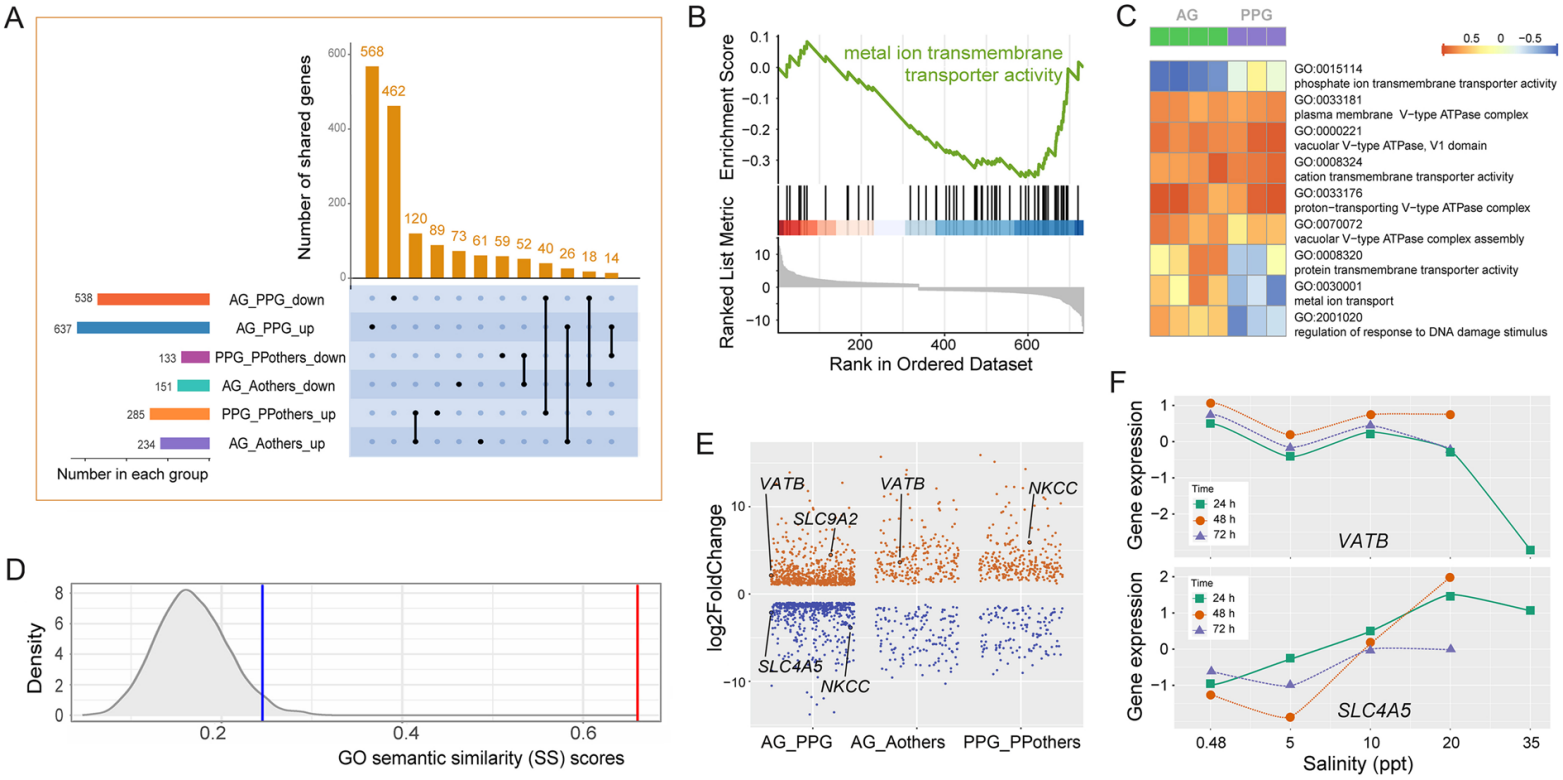
The genes and pathways show differential expression between *M.* *aosen* and *P.* *pacifica* gills. **A** Venn diagram of differentially expressed genes in the *M.* *aosen* gill (AG) relative to the *P.* *pacifica* gill (PPG), namely AG_PPG_up/down, *M.* *aosen* gill relative to other tissues (AG_Aothers_up/down), and *P.* *pacifica* gill relative to other tissues (PPG_PPothers_up/down). **B** Gene set enrichment analysis (GSEA) shows the correlations of genes involved in metal ion transmembrane transport activity between the two species. **C** Differentially expressed GO pathway related to osmoregulation identified by gene set variation analysis (GSVA). **D** The semantic similarity (SS) values of the GO terms show high similarity between the upregulated genes of these two species. **E** The shared DEGs in *M.* *aosen* gills relative to other tissues and *P.* *pacifica* gills, and in *P.* *pacifica* gills relative to other tissues and *M.* *aosen* gills. **F **Relative expression of *VATB* and *SLC4A5* under different salinity treatments validated by RT-qPCR. *Morinoia aosen* individuals that were not placed under a salinity gradient were treated as controls. More results of gene expression validation can be found in Supplementary Fig. S5

We first obtained 637 DEGs that were upregulated and 538 DEGs that were downregulated in *M. aosen* gills relative to *P. pacifica* (Fig. 4A). The GO enrichment analysis and GSEA based on these genes were performed to determine the gene sets that showed significant differences between the gills of *M. aosen* and *P. pacifica*. The results suggest that there was a difference in metal ion transmembrane transporter activity (GO:0046873) between species from marine vs. terrestrial habitats (Fig. 4B). To further illustrate the expression changes of each osmoregulation-related pathway, we performed GSVA and found that the vacuolar proton-transporting V-type H^+^-ATPases complex assembly (GO: 0070072), metal ion transport (GO:0030001) and protein transmembrane transporter activity (GO:0008320) showed higher expression in the *M. aosen* gills than that in the *P. pacifica* gills (Fig. 4C). In detail, the V-type H^+^-ATPases complex assembly pathway including five genes, namely *VATB*, *VATC*, *VATD*, *VATE*, and *VATF*, was upregulated. Furthermore, the gene encoding the sodium/hydrogen exchanger (Solute Carrier Family 9 Member A2, *SLC9A2*) was also upregulated. While the phosphate ion transmembrane transporter activity (GO: 0015114) and cation transmembrane transporter activity (GO:0008324) showed higher expression in *P. pacifica* gills, upregulated genes including *NKCC* and some solute carrier family genes, e.g., *SLC4A5*, *SLC41A1*, *SLC6A13*, were also detected. By conducting the Kyoto Encyclopedia of Genes and Genomes (KEGG) (http://www.genome.jp/kegg/) annotations of the upregulated genes in *P. pacifica* gills, several free amino acid metabolic pathways could be enriched, including “Valine, leucine and isoleucine degradation, dme00280”, “Histidine metabolism, dme00340”, “beta-Alanine metabolism, dme00410”, “Glycine, serine and threonine metabolism, dme00260”, and “Arginine and proline metabolism, dme00330” (Supplementary Table S5).

To explore whether the osmoregulatory organ of these two species exhibits some similarity in salinity adaptation, we compared the DEGs in gills relative to the other four tissues and obtained 385 DEGs (including 234 upregulated and 151 downregulated) in *M. aosen* gills, and 418 DEGs (including 285 upregulated and 133 downregulated) in *P. pacifica* gills. The semantic similarity values of the GO terms show high similarity between the upregulated genes of these two species (Fig. 4D; Supplementary Fig. S4). Among these genes, Na/K-ATPase was differentially upregulated in the gills relative to other tissues in both *M. aosen* and *P. pacifica.*

To determine the gene expression changes related to osmoregulation genes that were differentially expressed not only in gills relative to other tissues but also between species from separate habitats, we focused on the DEGs between gills of the two species and DEGs between the gills and other tissues (Fig. 4E). We obtained 26 upregulated and 18 downregulated genes in *M. aosen* gills relative to both other tissues and *P. pacifica* gills, and 40 upregulated and 14 downregulated genes in *P. pacifica* gills relative to both other tissues and *M. aosen* gills (Supplementary Table S6). According to the annotation of the upregulated genes in the gills of *P. pacifica*, *Chrna7* gene (Cholinergic Receptor Nicotinic Alpha 7 Subunit) encodes neurotransmitter-gated ion-channel and three genes related to inorganic ion transport, namely *NKCC* (Na^+^, K^+^, Cl^−^)*, SLC4A5* (Na^+^, HCO_3_^−^)*, SLC41A1* (Mg^2+^, Na^+^). For *M. aosen* gills, the two genes related to the ion channel were differentially expressed, i.e., *GLUCL* (Glutamate-gated Chloride Channels) which is related to the chloride channel was upregulated, and *KCNK1* (Potassium channel subfamily K member 1) which is related to the potassium channel and inorganic K^+^ transport was downregulated*.* Additionally, *SLC6A5, SLC6A6,* and *SLC6A13,* which belong to the SLC6 gene family were also downregulated.

We further used the salinity stress experiments and RT-qPCR to test the 14 DEGs (*VATB*, *VATC*, *VATD*, *VATE*, *VATF*, *VATG*, *VATH*, *VATO*, *VATS*, *VAT116 KDa*, *SLC4A5*, *SLC6A13*, *SLC41A1*, and *Chrna7*) expression changes under a salinity gradient (Fig. 4F). The decreased expression of all V-type H^+^-ATPases genes along the salinity gradient suggests that these genes are involved in the response to terrestrial habitats and are downregulated when the salinity increased (Supplementary Fig. S5). The increased expression of *SLC4A5*, *SLC41A1*, *SLC6A13,* and *Chrna7* with higher salinity suggests these genes are possibly involved in the high salinity stress response (Supplementary Fig. S5).

## Discussion

### Osmoregulatory genetic changes following sea–land transition

To detect the adaptive genetic changes of amphipods following the sea–land transition, we compared seven talitrid genomes and obtained a large number of orthologs, which provided a robust data basis for us to investigate genetic variations related to osmoregulation. Our reconstructed genomic phylogeny suggests that the terrestrial and coastal species originated from aquatic amphipods ~ 80.65 Ma, and then split ~ 14.33 Ma. These results support the ancient aquatic or marine origin of Talitridae and subsequent diversification between coastal and terrestrial species, which is congruent with previous research (Liu et al. 2023). Compared with the ancient aquatic-to-land transition of vertebrates (Wang et al. 2021), the recent divergence and dramatic habitat variation between terrestrial and coastal talitrids suggest this system is a suitable model for understanding the adaptive changes involved in the sea–land transition.

Terrestrial and coastal species live in habitats with different salinities, and osmotic tolerance is therefore a key process for successful land colonization. Gene family evolution and positive selection analyses indicate that at least three osmoregulatory gene families are significantly expanded, and many genes encoding transmembrane proteins associated with osmoregulation are positively selected, in terrestrial species. For instance, the gene family annotated as sodium/solute symporters, which consists of integral membrane proteins that use an existing sodium gradient to drive the transmembrane transport of various solutes, such as amino acids, ions, vitamins, or sugars, also plays an important role in osmoregulation in organisms that are subject to salinity fluctuation (Henriquez et al. 2021). Additionally, two RasGAP genes were positively selected and these play an important role in the physiological control of ion channel function (Pochynyuk et al. 2007; Yuan et al. 2017).

Adaptation of organisms to environmental change often leaves characteristic imprints of natural selection within their genomes. For example, the genes under selection tend to occur in genomic regions correlated with ion transport during the saline to freshwater transitions of the copepod *Eurytemora affinis* (Lee 2021; Stern and Lee 2020). Our results suggest that terrestrial species have evolved a cluster of genes with adaptive features that enhance water regulation and salt balance in response to the transition from sea to land. Similar adaptive regulation mechanisms can also be found in freshwater shrimps that have adapted to a narrow range of salinity compared with saltwater shrimps (Yuan et al. 2017). Because comparative genetic analysis relies heavily on the quality of genomes, the effects of the evolution of gene families and positively selected genes related to sea–land transition on this recent divergence model would have more thorough explanations using denser taxon sampling and higher quality genome assemblies and gene predictions.

### Gills function as the vital osmoregulatory organ

To detect the organ most closely linked to osmoregulation, we compared the specific and differential expression patterns of five tissues. The specific expression patterns of different tissues indicate their function, e.g., specifically expressed genes in the compound eyes related to vision, those in the nerves related to signal transduction, those in the pereopods related to muscle and cuticle, and those in the antennae related to sensory function. Our functional similarity comparisons suggest that the GO terms of specifically expressed genes in the gills do not significantly overlap with those in other tissues. This suggests that ion exchange and respiration are the primary functions of the gills, distinct from those of other tissues. Additionally, a large amount of DEGs in the gills relative to other tissues was enriched in pathways related to ion exchange and salinity balance. For example, Na/K-ATPase, which is located on the basolateral side of epithelial cells and was differentially upregulated in the gills relative to other tissues in both *M. aosen* and *P. pacifica*, is involved in transporting Na^+^ from epithelial cells to the hemolymph and is thought to be the principal driving force for ion uptake from the environment (Kirschner 2004; Lucu and Towle 2003; Towle and Kays 1986). All these results of gene expression and tissue comparison provide robust support for the assertion that the gills function as an osmoregulatory organ (Fig. 2).

WGCNA analyses indicate a highly correlated module associated with the gills, enriched in osmoregulation pathways such as proton and sodium ion transmembrane transport. Most high-weighted hub genes were V-type H^+^-ATPases, which are also related to H^+^ transport and have been reported to be responsible for acid–base balance, nitrogen excretion, and generating the ion flow in freshwater and extremely diluted environments (Tsai and Lin 2007; Weihrauch et al. 2001). The enriched osmoregulatory GO pathways in gill-related modules and key hub genes suggest that the core network may have relatively strong regulation for osmoregulation, and gills play a crucial role in maintaining osmoregulation homeostasis compared with other tissues.

The gills of crustaceans have long been considered a functional organ of osmoregulation and respiration, as seen in land crabs that have undergone sea–land transitions (Watson-Zink et al. 2021). The expression patterns and core gene set related to ion transport confirm that in talitrids, the gill is a vital osmoregulatory organ during sea–land transition. These findings shed valuable light on tissue function identification.

### Different regulatory mechanisms for the sea–land pair in response to salinity stress

**Fig. 5:**
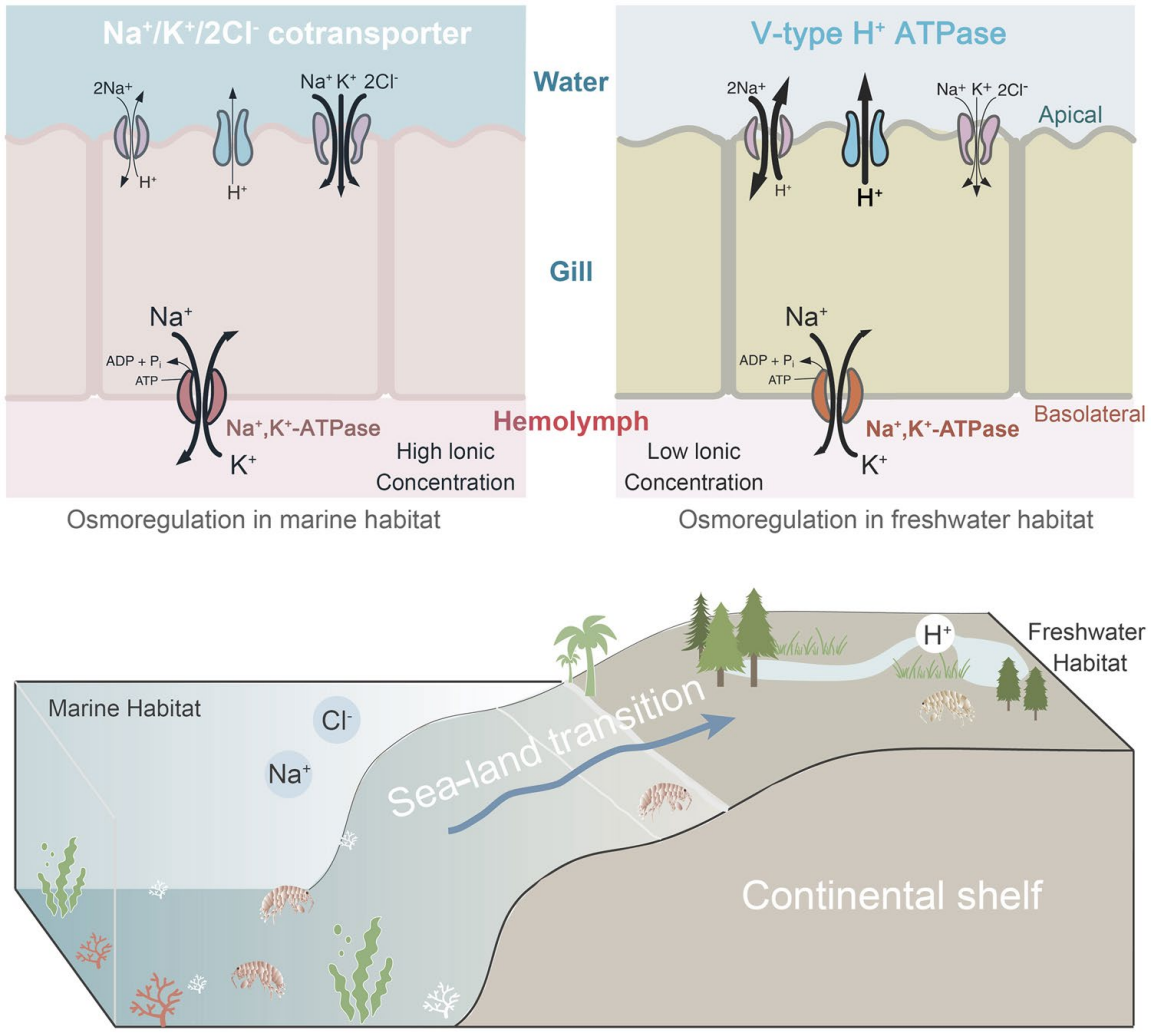
Hypothesized model of ion uptake across the gill showing a subset of putative ion transporters based on several DEGs in talitrids. In marine habitats, *NKCC* shows higher expression, so Na^+^ could simply diffuse into the gill epithelial cell. In land and freshwater habitats, Na^+^/H^+^ exchanger and V-type H^+^ ATPase show higher expression to pump H^+^ out of the cell and drive the transport of Na^+^ into the cell. In both habitats, Na^+^/K^+^-ATPase, which transports Na^+^ from the cell into the hemolymph, shows similar expression between habitats but higher expression relative to other tissues, suggesting the sea–land pair retains some common osmoregulation strategies depending on the habitat

Talitrids have colonized different ecosystems that are characterized by salinity variation. Due to the salinity differences between marine and inland habitats, as well as the varying hemolymph osmolality in *P. pacifica* and *M. aosen*, the DEGs in the gills of these species may provide insights into the mechanisms of salinity adaptation. Numerous genes were upregulated in the gills of sea–land species *M. aosen* and *P. pacifica* relative to other tissues. A high similarity in GO pathways for upregulated genes was observed between the two species. These results suggest that the gills employ similar osmoregulation mechanisms in both saline and freshwater habitats. For example, Na/K-ATPase, which is thought to be the principal driving force for ion uptake from the environment (Kirschner 2004; Lucu and Towle 2003), was upregulated in the gills of both species. Nevertheless, the different salinity conditions in marine vs terrestrial habitats require the organisms to adapt and maintain homeostasis through a variety of molecular, physiological, and ecological mechanisms. Given the recent divergence time and the similarity of gill morphology between these two species, we focused on the ion transporters related to DEGs to highlight their varying regulatory strategies in marine and freshwater habitats, rather than simply validating differences in ion transporters (Fig. 5). Given that many of these ion transporters perform other physiological functions in addition to ionic regulation, their evolutionary changes could result from responses to environmental variables other than salinity. Therefore, we demonstrated the genes and ion transporters related to salinity and added more possible models related to other physiological processes, such as intracellular pH regulation (Supplementary Fig. S6).

In the intertidal environment, coastal species face a higher habitat salinity and hemolymph osmolality than terrestrial species. Some genes related to inorganic ion transport were upregulated relative to those of terrestrial species. For example, *NKCC* is upregulated in *P. pacifica*, leading to Na^+^ and Cl^−^ flow across an apical Na^+^/K^+^/2 Cl^−^ symporter driven by the inward Na^+^ gradient, supplemented by apical K^+^ channels that recycle K^+^, hyperpolarizing the apical membrane (McNamara and Faria 2012). There is some evidence for the participation of the *NKCC* in salt secretion of some crustacean species across the gill epithelium, which is upregulated in the gills of the freshwater shrimp *Macrobrachium australiense* when challenged by an increase in salinity (Maraschi et al. 2021).

In the inland habitats, terrestrial talitrids live near lakes and riverbanks, and these freshwater habitats are much more stable in terms of salinity compared to the coast. Genes encoding V-type H^+^-ATPases and Na^+^/H^+^ exchanger are upregulated in *M. aosen*. The V-type H^+^-ATPases play critical roles in ion regulation by actively transporting intracellular protons out of the cell to generate a transmembrane electrical potential that allows extracellular Na^+^ to flow into the cells via Na^+^ channels and the Na^+^/H^+^ exchanger (Lee et al. 2011; Thabet et al. 2017). The V-type H^+^-ATPases not only function as the core network in the gills but are also upregulated in terrestrial talitrids, suggesting that the V-type H^+^-ATPases are a crucial component for the transport of ions against steep concentration gradients in freshwater habitats. Free amino acids are known to play an important role in adaptation to low salinity in crustaceans (Yao et al. 2021). Some genes involved in amino acid, nutrient, or other kinds of osmolyte transport across the plasma membrane (e.g., *SLC6A13*) show decreased expression in *M. aosen* gills, suggesting the potential role of free amino acids in the maintenance of cell volume and adaptation to terrestrial habitats (Bröer and Gether 2012).

Gene expression validation using RT-qPCR suggests upregulated genes in the terrestrial talitrids *M. aosen* (mainly V-type H^+^-ATPases genes) showed gradually decreased expression trends, while some upregulated genes in coastal species *P. pacifica* (*SLC4A5*, *SLC6A13*, *SLC41A1*, *Chrna7*) showed gradually increased expression trends when facing increasing salinity. We found that plastic expression changes in salinity stress experiments and evolutionary responses in gene expression between marine and land talitrids were highly positively correlated. The plastic response could potentially facilitate the adaptive response. A strong beneficial plastic response to novel conditions, such as the increased expression of a critical ion transporter in freshwater, can help organisms survive upon arriving in a new habitat (Lee et al. 2021).

The fluctuating gene expressions along salinity changes not only reflect the genetic differences in the sea–land adaptation mechanisms but also suggest that these species and their expression level of osmoregulatory genes are potential biomarkers for salinity changes in biological monitoring. They could serve as a predictor of how ecosystems respond to disturbance or the presence of a stressor, and allow the integration of responses to current, past, or future environmental conditions by adding a temporal component that corresponds to their life span or residence time.

## Conclusions

Understanding how natural populations respond to changing environmental conditions is a fundamental research focus in ecology and has profound importance in conservation biology. Our results have shown that the gills are important osmoregulatory organs and provide clear signs of an organism’s differential response to changes in salinity. Gene expression changes involved in ion exchange in the gills could contribute to salinity adaptation during the evolutionary transition from saline to land habitats. The salinity levels of saltwater and freshwater habitats are impacted by human demands for freshwater, climate change‐driven precipitation variability, and extreme weather. The diverse habitats of talitrids and clear knowledge of their genetic response to salinity provide a new model for biological monitoring and help to make conservation decisions in different ecosystems under changing climates.

## Supplementary Information

Below is the link to the electronic supplementary material.

Supplementary file1 (DOCX 1128 KB)

## Data Availability

All raw RNA-seq data generated in this study are deposited into the NCBI SRA under the accession number given PRJNA1210989 and PRJNA938803. All other data are available in the supplementary materials.
