# Supplementary material for: Osmoregulatory evolution of gills promoted salinity adaptation following the sea–land transition of crustaceans
Source: Mar Life Sci Technol. 2025 May 15;7(2):205–17. doi: 10.1007/s42995-025-00298-6 (PMC12102416; doi:10.1007/s42995-025-00298-6)
Supplement: Supplementary file 1 — Supplementary file1 (DOCX 1128 KB) [file 42995_2025_298_MOESM1_ESM.docx]

# Supplementary figures


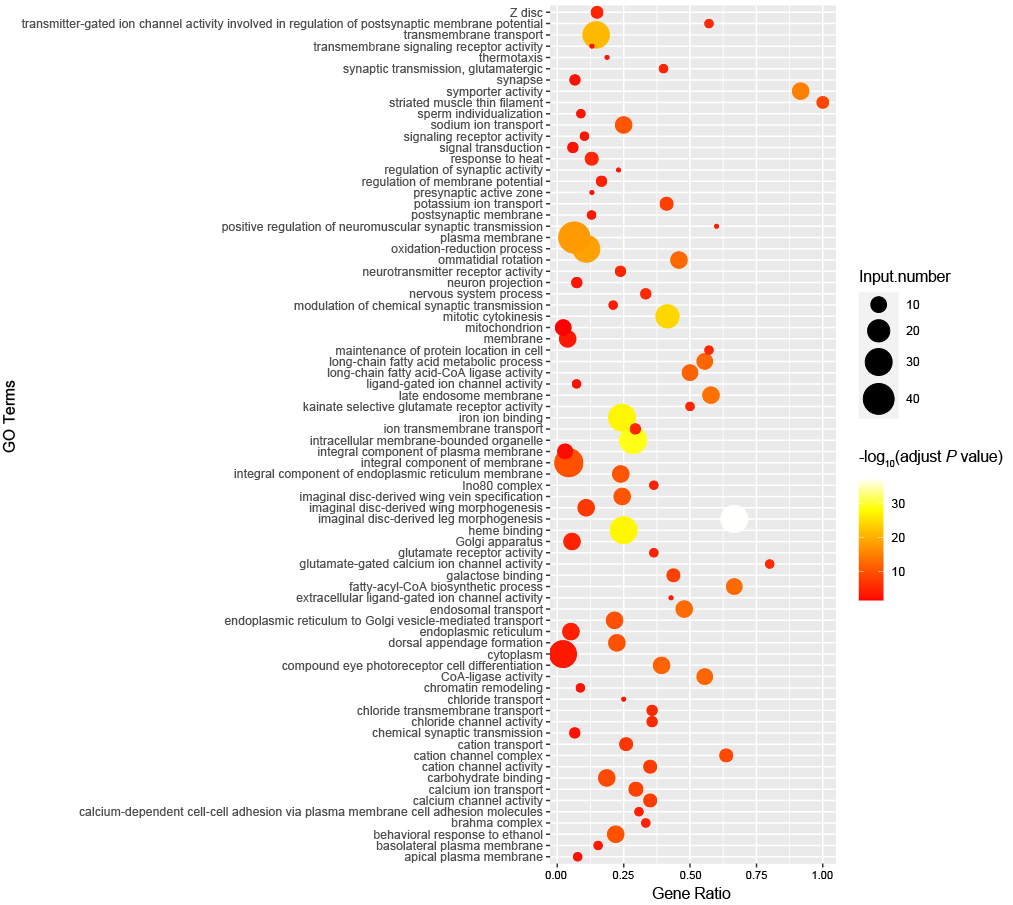


**Fig. S1 The GO enrichment results of significantly expanded gene families in terrestrial talitrids compared with coastal talitrids.**


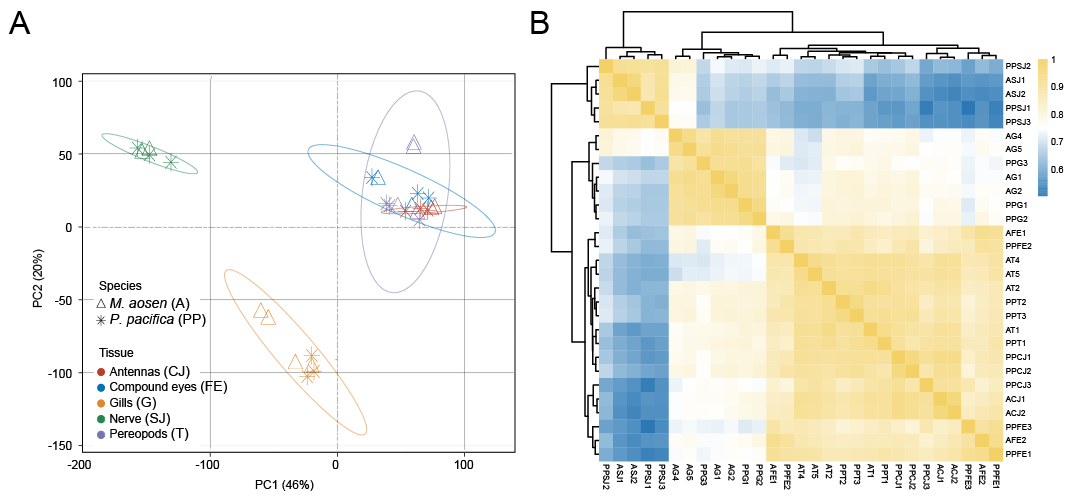


**Fig. S2 Principal component analysis (A) and Symmetrical heat map of Spearman’s correlation coefficients (B) show that data for the gills are clearly separated from those of other tissues.** Samples are represented with abbreviations of species+tissues+samples number. e.g., AG1: *Morinoia aosen* gills sample1.


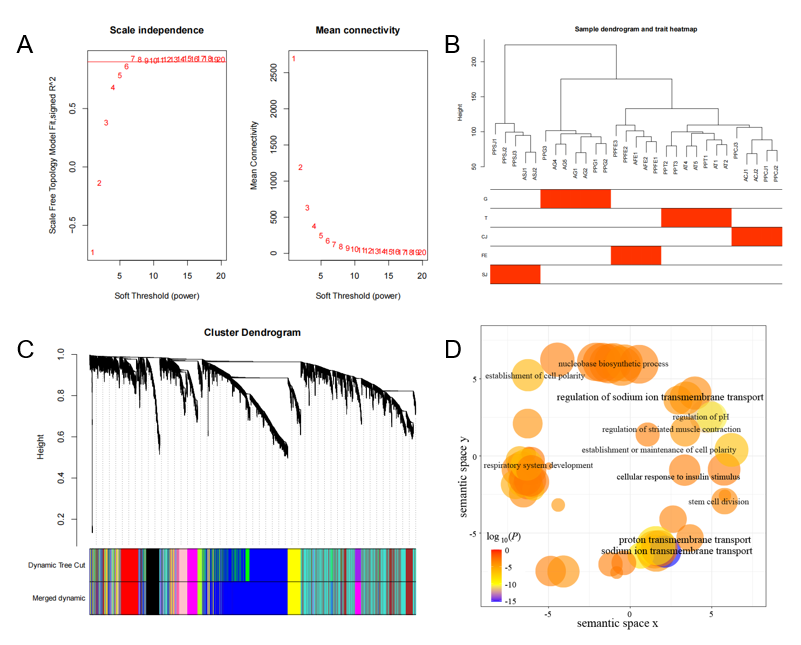


**Fig. S3 The Weighted gene Co-expression analysis (WGCNA) for five tissues in *Morinoia aosen* and *Platorchestia pacifica*.** **A** Analysis of soft-thresholding powers based on scale independence (left) and mean connectivity (right). Soft threshold of 6 was selected. **B** Sample cluster analysis. All the same tissues cluster together, indicating reasonable biological repetitions. **C** The cluster dendrogram of genes. Modules considered with high similarity were merged. **D** Enriched GO terms of the of magenta module associated with gill genes. Bubble size represents the number of annotations for a certain GO term, and color indicates the corrected enrichment *P*-value on a log_10_ scale.


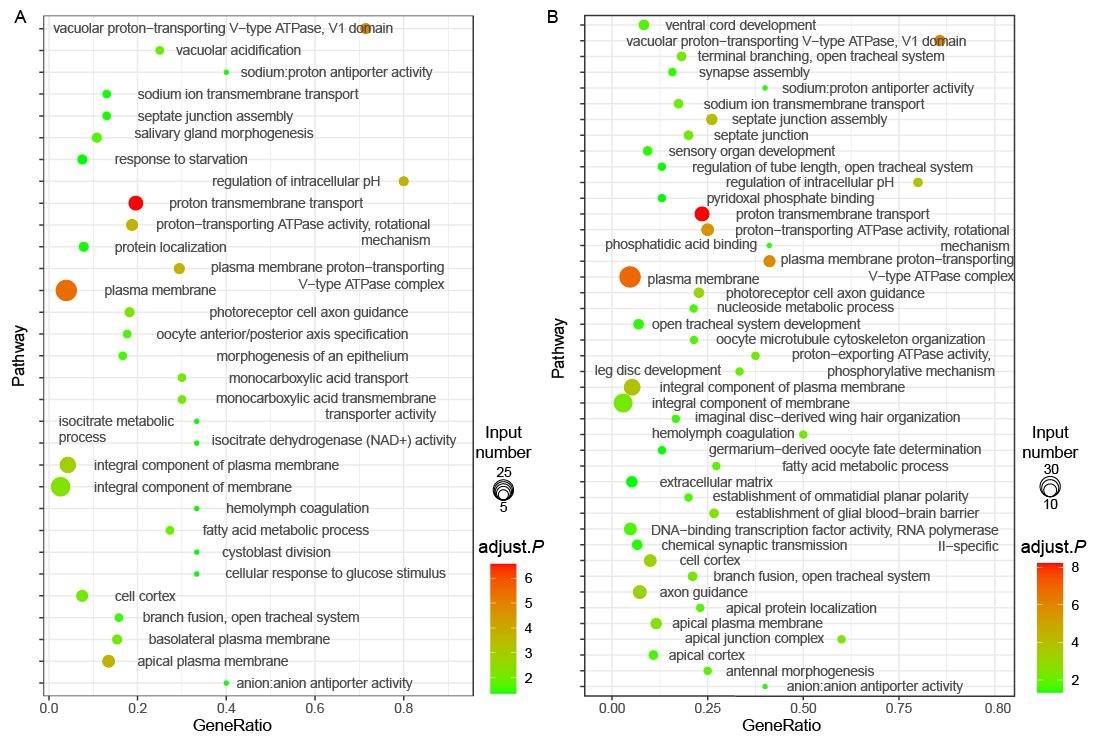


**Fig. S4** **GO enrichment of** **upregulated genes in gills** **relative to other tissues in *Morinoia* *aosen* and *Platorchestia pacifica*. A** GO enrichment results of upregulated genes in *M. aosen* gills relative both to other tissues and to *P. pacifica* gills. **B** GO enrichment results of upregulated genes in *P. pacifica* gills relative both to other tissues and to *M. aosen* gills.


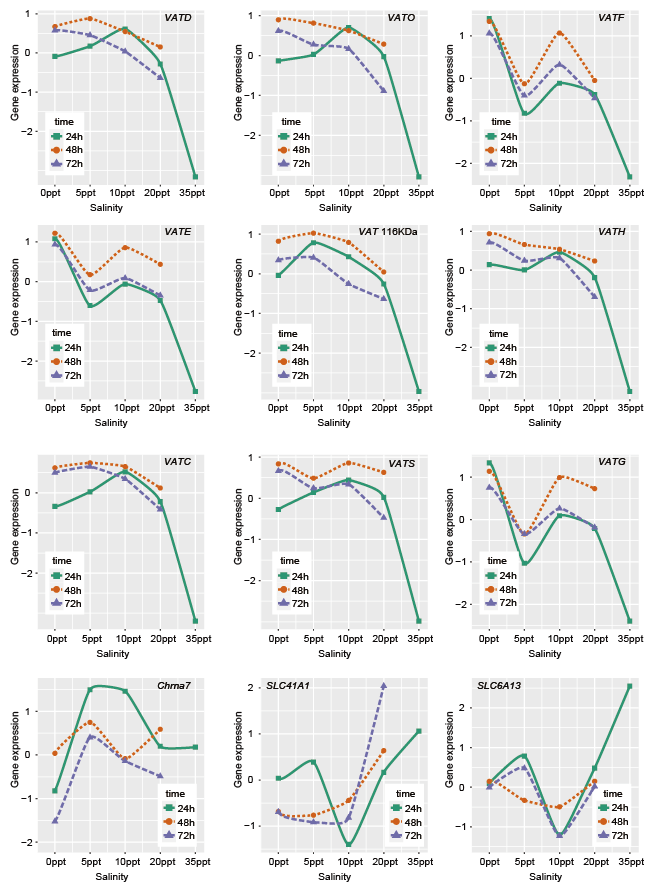


**Fig. S5. The DEGs expression changes in *Morinoia aosen* under the gradient salinity acclimation validated by the RT-qPCR.** *M. aosen* that were not treated were treated as a control.


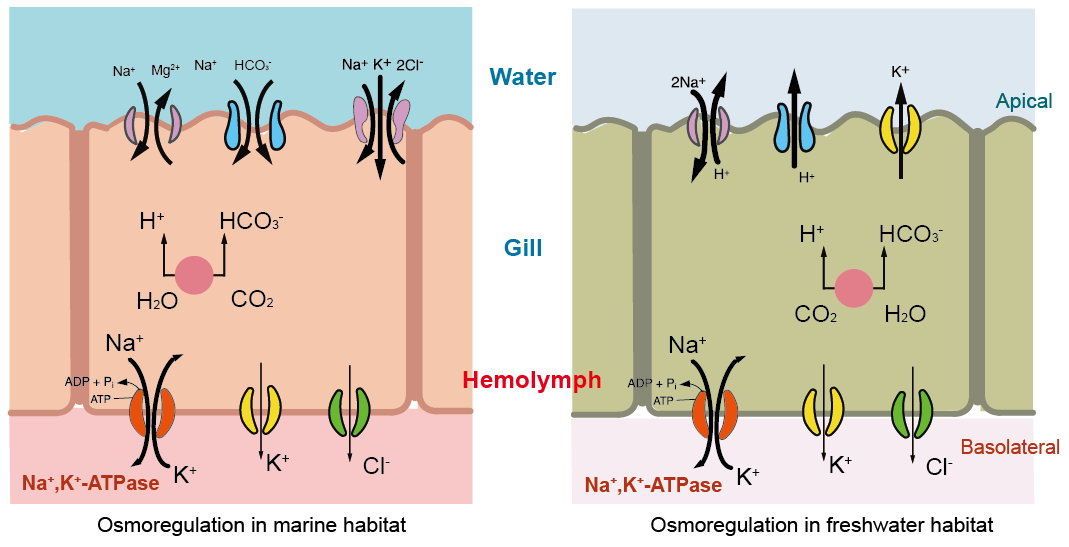


**Fig. S6 Other possible models related to ion transport and pH regulation in talitrids based on several DEGs.** In marine habitats, genes related to inorganic ion transport, i.e., *NKCC* (Na^+^, K^+^, Cl^−^), *SLC4A5* (Na^+^, HCO_3_^−^), and *SLC41A1* (Mg^2+^, Na^+^), were upregulated. In freshwater habitats, V-type H^+^-ATPases and *SLC9A2* (sodium/hydrogen exchanger) were upregulated, while *KCNK1* (Potassium channel subfamily K member 1), which is related to the potassium channel and inorganic K^+^ transport, was downregulated. Na^+^/K^+^-ATPase, which transports Na^+^ from the cell into the hemolymph, shows similar expression between habitats but higher expression relative to other tissues. CO_2_ hydration by cytosolic carbonic anhydrase generates H^+^ and HCO_3_^−^, perhaps originally coupled to intracellular pH regulation.

# Supplementary Tables

Table S1. BUSCO-based assessment (arthropoda_odb10) of genome coding sequence used in this study.

| Type | | Complete BUSCOs (C) | Complete and single-copy BUSCOs (S) | Complete and duplicated BUSCOs (D) | Fragmented BUSCOs (F) | Missing BUSCOs (M) |
| --- | --- | --- | --- | --- | --- | --- |
| *Morinoia aosen* (GCA_030386875.1) | Number | 957 | 873 | 84 | 16 | 40 |
|  | Rate（%） | 94.5 | 86.2 | 8.3 | 1.6 | 3.9 |
| *Platorchestia*  *hallaensis*  (GCA_014220935.1) | Number | 743 | 730 | 13 | 132 | 138 |
|  | Rate（%） | 73.4 | 72.1 | 1.3 | 13.0 | 13.6 |
| *Platorchestia pacifica* (This study) | Number | 905 | 800 | 105 | 53 | 55 |
|  | Rate（%） | 89.4 | 79.0 | 10.4 | 5.2 | 5.4 |
| *Ochestia grillus*  (GCA_014899125.1) | Number | 630 | 624 | 6 | 203 | 180 |
|  | Rate（%） | 62.2 | 61.6 | 0.6 | 20 | 17.8 |
| *Trinorchestia longiramus*  (GCA_006783055.1) | Number | 860 | 827 | 33 | 84 | 69 |
|  | Rate（%） | 84.9 | 81.6 | 3.3 | 8.3 | 6.8 |
| *Parhyale*  *hawaiensis*  (GCA_001587735.2) | Number | 970 | 953 | 17 | 20 | 23 |
|  | Rate（%） | 95.8 | 94.1 | 1.7 | 2.0 | 2.2 |
| *Hyalella azteca*  (GCA_000764305.4) | Number | 955 | 865 | 90 | 27 | 31 |
|  | Rate（%） | 94.3 | 85.4 | 8.9 | 2.7 | 3.0 |

Table S2. Positively selected genes with *P*-values. Two terrestrial species (*Morinoia aosen* and *Platorchestia hallaensis*) were set as foreground branches.

| **GeneID** | **genename** | **lnL** | **lnL0** | **P** | **FDR** |
| --- | --- | --- | --- | --- | --- |
| gmmseq476G001290.2 | *TUT4* | -6169.64 | -6234.38 | 5.32E-30 | 1.2291E-26 |
| gmmseq204G001400.1 | *TROP* | -2976.12 | -3008.9 | 5.64E-16 | 6.51861E-13 |
| gmmseq142G001080.1 | *-* | -5039.74 | -5067.96 | 5.77E-14 | 4.44799E-11 |
| gmmseq385G000810.3 | *RER1* | -1086.59 | -1112.26 | 7.79E-13 | 4.5055E-10 |
| gmmseq503G000010.1 | *MTX1* | -2377.78 | -2402.69 | 1.69E-12 | 7.83181E-10 |
| gmmseq115G001700.1 | *MYOB1* | -4146.23 | -4168.52 | 2.44E-11 | 9.40654E-09 |
| gmmseq739G000100.1 | *-* | -4365.78 | -4386.9 | 8.07E-11 | 2.66577E-08 |
| gmmseq207G000050.1 | *FLOT1* | -2170.37 | -2189.86 | 4.27E-10 | 1.2337E-07 |
| gmmseq598G000540.1 | *STEA4* | -4910.02 | -4928.96 | 7.54E-10 | 1.93679E-07 |
| gmmseq589G000740.1 | *INCE* | -8740.36 | -8757.37 | 5.43E-09 | 1.25644E-06 |
| gmmseq792G000080.1 | *FANCM* | -15931 | -15947.3 | 1.19E-08 | 2.50618E-06 |
| gmmseq602G000440.1 | *-* | -16193.1 | -16208.8 | 2.05E-08 | 3.94172E-06 |
| gmmseq399G001060.1 | *-* | -4797.02 | -4812.31 | 3.23E-08 | 5.73698E-06 |
| gmmseq396G000690.1 | *PEG3* | -8646.48 | -8661.74 | 3.31E-08 | 5.47195E-06 |
| gmmseq163G000350.1 | *PKD1* | -18017.2 | -18032 | 5.69E-08 | 8.77168E-06 |
| gmmseq402G000680.1 | *RAB3GAP2* | -3925.55 | -3940.15 | 6.53E-08 | 9.43801E-06 |
| gmmseq856G000020.1 | *KHDC3* | -7737.53 | -7751.78 | 9.44E-08 | 1.28438E-05 |
| gmmseq117G000640.1 | *CENPE* | -30916.4 | -30930.3 | 1.37E-07 | 1.75995E-05 |
| gmmseq198G000300.1 | *SYCP2* | -18491.1 | -18504.9 | 1.41E-07 | 1.71254E-05 |
| gmmseq245G002290.1 | *-* | -4325.23 | -4338.9 | 1.71E-07 | 1.9792E-05 |
| gmmseq19G000050.1 | *YFL067W* | -1288.44 | -1301.71 | 2.6E-07 | 2.86064E-05 |
| gmmseq556G000200.2 | *CDAN1* | -12520.4 | -12532.7 | 7.36E-07 | 7.7322E-05 |
| gmmseq65G000430.1 | *PRP2* | -20141.1 | -20152.8 | 1.28E-06 | 0.000128716 |
| gmmseq740G000680.1 | *ZN626* | -9144.81 | -9156.42 | 1.44E-06 | 0.000139105 |
| gmmseq754G000210.1 | *SMYD4* | -7986.04 | -7997.55 | 1.59E-06 | 0.000147367 |
| gmmseq271G000260.1 | *KIF4B* | -6573.34 | -6584.06 | 3.65E-06 | 0.000324721 |
| gmmseq509G000130.1 | *REN3A* | -8095.19 | -8105.9 | 3.7E-06 | 0.000316628 |
| gmmseq129G001270.1 | *-* | -2908.47 | -2919.09 | 4.05E-06 | 0.000334129 |
| gmmseq354G000920.2 | *DIAP2* | -5955.12 | -5965.73 | 4.11E-06 | 0.000327655 |
| gmmseq736G000120.1 | *HPF1* | -2619.21 | -2629.69 | 4.73E-06 | 0.000364678 |
| gmmseq733G000420.1 | *IPPK* | -10616.6 | -10627 | 5.05E-06 | 0.000376897 |
| gmmseq497G000940.1 | *MCAF1* | -8892.16 | -8902.56 | 5.14E-06 | 0.000371694 |
| gmmseq791G000100.1 | *FA5* | -11594.5 | -11604.7 | 6.51E-06 | 0.000456158 |
| gmmseq564G000730.1 | *-* | -7799.55 | -7809.62 | 7.23E-06 | 0.000491827 |
| gmmseq54G000190.1 | *METL4* | -2835.89 | -2845.95 | 7.31E-06 | 0.000482711 |
| gmmseq422G000480.1 | *-* | -8852.77 | -8862.75 | 7.87E-06 | 0.000505289 |
| gmmseq22G000160.1 | *PTH1R* | -4157.2 | -4167.1 | 8.62E-06 | 0.000538903 |
| gmmseq312G000190.1 | *TRM13* | -5023.99 | -5033.77 | 9.69E-06 | 0.000589639 |
| gmmseq69G000260.1 | *ABHEB* | -1546.28 | -1555.61 | 1.56E-05 | 0.000922268 |
| gmmseq482G000510.1 | *MOCOS* | -6173.84 | -6183.02 | 1.82E-05 | 0.00105323 |
| gmmseq204G000220.1 | *-* | -7766.76 | -7775.91 | 1.88E-05 | 0.001061745 |
| gmmseq250G000160.1 | *-* | -1887.03 | -1895.96 | 2.38E-05 | 0.001311124 |
| gmmseq22G000020.1 | *-* | -11443.7 | -11452.6 | 2.63E-05 | 0.001413488 |
| gmmseq486G000260.1 | *CCNY* | -8204.35 | -8213.07 | 2.95E-05 | 0.001549621 |
| gmmseq48G000240.1 | *SDR9C7* | -5760.5 | -5769.07 | 3.5E-05 | 0.001796656 |
| gmmseq476G001610.1 | *OCRL* | -2105.71 | -2114.13 | 4.06E-05 | 0.002042228 |
| gmmseq574G000510.1 | *FAR1* | -2449.09 | -2457.43 | 4.39E-05 | 0.002160295 |
| gmmseq51G001220.1 | *CTHB1* | -3301.29 | -3309.6 | 4.55E-05 | 0.002193087 |
| gmmseq750G000150.1 | *NO12B* | -5250.58 | -5258.85 | 4.77E-05 | 0.002248523 |
| gmmseq407G000170.1 | *WHITE* | -10212.5 | -10220.6 | 5.32E-05 | 0.002459448 |
| gmmseq518G000080.1 | *RPGRH* | -9496.82 | -9504.99 | 5.33E-05 | 0.002414462 |
| gmmseq585G001090.1 | *PTGR1* | -2288.43 | -2296.54 | 5.63E-05 | 0.002501976 |
| gmmseq466G000400.1 | *NIPA* | -5889.95 | -5898.06 | 5.63E-05 | 0.002455404 |
| gmmseq539G000140.1 | *HEM3* | -2274.39 | -2282.45 | 5.93E-05 | 0.002539304 |
| gmmseq304G000100.1 | *-* | -12044 | -12052 | 5.94E-05 | 0.002495653 |
| gmmseq508G000200.1 | *-* | -4251.96 | -4259.85 | 7.14E-05 | 0.002948249 |
| gmmseq469G000750.1 | *CFDP1* | -2323.99 | -2331.77 | 7.97E-05 | 0.003233095 |
| gmmseq258G000170.1 | *TAB1* | -2349.63 | -2357.38 | 8.27E-05 | 0.003296704 |
| gmmseq593G000250.1 | *C2CD5* | -5561.48 | -5569.19 | 8.56E-05 | 0.003353271 |
| gmmseq252G000500.1 | *CBPB* | -8589.17 | -8596.88 | 8.64E-05 | 0.00332795 |
| gmmseq68G000280.1 | *XPO5* | -5086.7 | -5094.37 | 9.01E-05 | 0.00341366 |
| gmmseq143G000360.1 | *TMC7* | -1655.39 | -1663.01 | 9.4E-05 | 0.003506872 |
| gmmseq132G001540.1 | *KIF3B* | -9893.92 | -9901.49 | 9.98E-05 | 0.003663652 |
| gmmseq48G000260.1 | *THUM3* | -3909.17 | -3916.72 | 0.000102 | 0.003672587 |
| gmmseq497G001380.1 | *FP1* | -10123.4 | -10130.9 | 0.000103 | 0.003661058 |
| gmmseq91G000270.1 | *ARRD3* | -2429.54 | -2436.92 | 0.000122 | 0.004286254 |
| gmmseq68G000390.2 | *DTX2* | -7725.51 | -7732.85 | 0.000126 | 0.004363029 |
| gmmseq467G000440.1 | *THADA* | -12097.6 | -12104.9 | 0.000132 | 0.004472617 |
| gmmseq274G001970.1 | *PTPRC* | -7232.08 | -7239.32 | 0.000142 | 0.004744282 |
| gmmseq85G000080.1 | *CLRN3* | -2993.61 | -3000.81 | 0.000148 | 0.004874586 |
| gmmseq16G000180.1 | *RESIL* | -7696.07 | -7703.18 | 0.000162 | 0.005279997 |
| gmmseq255G000870.1 | *APLF* | -5532.22 | -5539.28 | 0.000171 | 0.005483624 |
| gmmseq85G000360.1 | *NXN* | -3999.95 | -4006.93 | 0.000186 | 0.005902277 |
| gmmseq39G000200.1 | *OCTL* | -4264.67 | -4271.65 | 0.000187 | 0.005843087 |
| gmmseq589G000660.1 | *-* | -1613.18 | -1620.11 | 0.000197 | 0.00608646 |
| gmmseq134G000030.1 | *BMAL1* | -4123.19 | -4129.95 | 0.000236 | 0.007166754 |
| gmmseq736G000070.1 | *PP12C* | -9338.12 | -9344.87 | 0.000239 | 0.007178669 |
| gmmseq736G000220.1 | *-* | -2522.15 | -2528.72 | 0.000289 | 0.008571072 |
| gmmseq376G000250.1 | *BMI1B* | -6502.18 | -6508.71 | 0.0003 | 0.008772954 |
| gmmseq385G001500.2 | *ZN628* | -11106.4 | -11112.9 | 0.000306 | 0.008832882 |
| gmmseq824G000040.1 | *-* | -12396.6 | -12403.1 | 0.000333 | 0.009495244 |
| gmmseq644G000960.1 | *ZN236* | -3253.87 | -3260.27 | 0.000348 | 0.009804966 |
| gmmseq274G000830.1 | *DNMBP* | -8029.92 | -8036.31 | 0.000352 | 0.009816426 |
| gmmseq68G000590.1 | *GAGA* | -6764.31 | -6770.63 | 0.000377 | 0.010376675 |
| gmmseq101G000280.1 | *LACH* | -713 | -719.311 | 0.000381 | 0.010369444 |
| gmmseq248G000320.1 | *MIPT3* | -2694.25 | -2700.53 | 0.000394 | 0.01058392 |
| gmmseq247G001190.2 | *PDE* | -4237.45 | -4243.66 | 0.000426 | 0.011323406 |
| gmmseq206G000510.1 | *-* | -3855.53 | -3861.67 | 0.000454 | 0.011924704 |
| gmmseq607G000160.1 | *-* | -1583.25 | -1589.38 | 0.000461 | 0.011978626 |
| gmmseq93G000140.1 | *RBM5A* | -5110.33 | -5116.46 | 0.000463 | 0.011889526 |
| gmmseq336G000220.1 | *ESCA* | -5293.24 | -5299.37 | 0.000464 | 0.011776075 |
| gmmseq392G000170.1 | *-* | -1216.81 | -1222.93 | 0.00047 | 0.011822017 |
| gmmseq607G000560.1 | *ZFP26* | -5420.46 | -5426.55 | 0.000479 | 0.011901815 |
| gmmseq855G000080.1 | *-* | -1038.57 | -1044.62 | 0.000502 | 0.012355498 |
| gmmseq768G000170.1 | *TOPRS* | -7948.39 | -7954.43 | 0.000512 | 0.012464361 |
| gmmseq250G000720.1 | *U518* | -4355.53 | -4361.47 | 0.000565 | 0.013610744 |
| gmmseq476G000940.1 | *PLBLB* | -3257.44 | -3263.36 | 0.000577 | 0.013749975 |
| gmmseq206G000500.1 | *-* | -2558.42 | -2564.32 | 0.000596 | 0.014049495 |
| gmmseq526G000650.1 | *-* | -898.629 | -904.428 | 0.00066 | 0.015411078 |
| gmmseq410G000410.1 | *CU27* | -4483.62 | -4489.41 | 0.000663 | 0.015335666 |
| gmmseq569G000150.1 | *GEMI5* | -14163.8 | -14169.6 | 0.000669 | 0.015303872 |
| gmmseq274G000530.1 | *-* | -11389.6 | -11395.4 | 0.000679 | 0.015391509 |
| gmmseq618G000210.1 | *-* | -738.407 | -744.144 | 0.000706 | 0.015842767 |
| gmmseq385G000280.1 | *-* | -2379.84 | -2385.5 | 0.000766 | 0.017036077 |
| gmmseq887G000020.1 | *-* | -9660.24 | -9665.88 | 0.000776 | 0.017094345 |
| gmmseq274G000300.1 | *RBMX2* | -2849.69 | -2855.28 | 0.000829 | 0.018078665 |
| gmmseq735G000280.1 | *-* | -1873.35 | -1878.9 | 0.000864 | 0.018665879 |
| gmmseq132G002120.1 | *-* | -830.479 | -836.026 | 0.000866 | 0.018547194 |
| gmmseq115G000810.1 | *-* | -1033.61 | -1039.12 | 0.000901 | 0.019120161 |
| gmmseq826G000490.1 | *-* | -515.532 | -521.004 | 0.000939 | 0.019732605 |
| gmmseq206G001410.1 | *MTH2* | -13707 | -13712.5 | 0.000955 | 0.019882193 |
| gmmseq142G000860.1 | *OPLA* | -3435.02 | -3440.44 | 0.000987 | 0.020381716 |
| gmmseq115G000890.2 | *IQGA1* | -14910.7 | -14916.1 | 0.001012 | 0.020698604 |
| gmmseq505G000370.1 | *MACOI* | -3214.67 | -3220.06 | 0.001034 | 0.020960371 |
| gmmseq591G000650.1 | *TM169* | -2210.46 | -2215.8 | 0.001078 | 0.021667631 |
| gmmseq771G000280.1 | *SANT* | -1441.12 | -1446.47 | 0.001082 | 0.021562487 |
| gmmseq794G000320.1 | *TRFM* | -2712.79 | -2718.12 | 0.001092 | 0.021575874 |
| gmmseq21G000070.1 | *CycB3* | -603.541 | -608.856 | 0.001112 | 0.021788601 |
| gmmseq791G000050.1 | *RESIL* | -2119.11 | -2124.41 | 0.001133 | 0.022011877 |
| gmmseq743G000290.1 | *AASD1* | -2307.35 | -2312.64 | 0.001146 | 0.022072958 |
| gmmseq577G000340.1 | *-* | -7441.53 | -7446.81 | 0.001158 | 0.022124333 |
| gmmseq115G001150.1 | *MTTF* | -4080.41 | -4085.67 | 0.001176 | 0.022287419 |
| gmmseq8G000110.1 | *NFH* | -1965.88 | -1971.09 | 0.001247 | 0.023444169 |
| gmmseq735G000390.1 | *FHOD3* | -5912.24 | -5917.43 | 0.001269 | 0.023651923 |
| gmmseq67G000810.1 | *EIF3A* | -7591.9 | -7597.09 | 0.001281 | 0.023688259 |
| gmmseq175G000240.1 | *-* | -4242.32 | -4247.5 | 0.001285 | 0.023570857 |
| gmmseq458G000010.1 | *RUSD2* | -1186.29 | -1191.45 | 0.001316 | 0.02395399 |
| gmmseq181G000450.1 | *TTN1* | -21068.1 | -21073.2 | 0.001331 | 0.024045098 |
| gmmseq225G000090.1 | *PPAF2* | -1475.92 | -1481.05 | 0.001357 | 0.024326542 |
| gmmseq407G000360.1 | *PCE* | -1954.32 | -1959.42 | 0.001401 | 0.024917037 |
| gmmseq793G000050.1 | *-* | -1263.5 | -1268.55 | 0.001489 | 0.026286278 |
| gmmseq129G001770.1 | *-* | -5373.53 | -5378.54 | 0.001554 | 0.027222796 |
| gmmseq183G000160.1 | *-* | -2651.35 | -2656.36 | 0.001559 | 0.027096977 |
| gmmseq143G000420.1 | *NLRC4* | -6571.98 | -6576.98 | 0.001569 | 0.027069876 |
| gmmseq480G000580.1 | *LIGO1* | -3982.04 | -3987.04 | 0.001569 | 0.026871168 |
| gmmseq385G002180.1 | *NDUBA* | -958.088 | -963.084 | 0.001572 | 0.026718178 |
| gmmseq733G000200.1 | *CAD96* | -8183.3 | -8188.27 | 0.001605 | 0.02708168 |
| gmmseq579G000450.1 | *GLRA3* | -3754.19 | -3759.16 | 0.001627 | 0.027255454 |
| gmmseq421G001860.1 | *GCM2* | -3830.32 | -3835.28 | 0.001639 | 0.027261062 |
| gmmseq345G001520.1 | *-* | -2739.3 | -2744.25 | 0.001648 | 0.027210377 |
| gmmseq206G002080.1 | *-* | -3240.87 | -3245.82 | 0.00166 | 0.027211501 |
| gmmseq604G000300.1 | *DDX51* | -3331.29 | -3336.24 | 0.001666 | 0.027131992 |
| gmmseq164G000740.1 | *CELR2* | -6119.06 | -6123.99 | 0.001687 | 0.027276805 |
| gmmseq3G000220.1 | *NRG* | -1380.31 | -1385.24 | 0.001689 | 0.02711042 |
| gmmseq132G001910.1 | *-* | -3903 | -3907.92 | 0.001714 | 0.027326728 |
| gmmseq452G000110.1 | *COG8* | -1246.93 | -1251.82 | 0.001757 | 0.027826399 |
| gmmseq405G000160.1 | *NFH* | -7376.19 | -7381.08 | 0.001767 | 0.027796798 |
| gmmseq20G000200.1 | *-* | -14865.6 | -14870.5 | 0.001808 | 0.028237452 |
| gmmseq203G000180.1 | *AGK* | -1721.12 | -1725.94 | 0.00191 | 0.029643318 |
| gmmseq193G000060.1 | *SAP* | -2008.5 | -2013.31 | 0.001934 | 0.02980207 |
| gmmseq644G000610.1 | *HSP70* | -6100.8 | -6105.61 | 0.001934 | 0.029615056 |
| gmmseq372G001270.1 | *-* | -5287.43 | -5292.18 | 0.002048 | 0.031149155 |
| gmmseq564G000950.1 | *UTP6* | -4651.91 | -4656.66 | 0.00207 | 0.031274907 |
| gmmseq206G000120.1 | *MTAP2* | -1199.33 | -1204.01 | 0.002203 | 0.033073285 |
| gmmseq776G000270.1 | *ZN678* | -14152 | -14156.7 | 0.002232 | 0.033293665 |
| gmmseq447G000270.1 | *EMC7* | -1034.04 | -1038.7 | 0.002284 | 0.033849255 |
| gmmseq115G001870.1 | *ZN367* | -9021.63 | -9026.25 | 0.002373 | 0.034946203 |
| gmmseq123G000420.1 | *-* | -2998.15 | -3002.77 | 0.002377 | 0.034782838 |
| gmmseq33G001170.2 | *Ppp6r3* | -3036.24 | -3040.85 | 0.002382 | 0.034643151 |
| gmmseq823G000130.1 | *PRD10* | -2399.07 | -2403.68 | 0.002401 | 0.034691154 |
| gmmseq198G000410.1 | *RECQ4* | -14369.8 | -14374.4 | 0.002453 | 0.035226681 |
| gmmseq370G000460.1 | *-* | -11231.2 | -11235.7 | 0.002564 | 0.036594161 |
| gmmseq65G000160.2 | *TGIF1* | -7168.69 | -7173.21 | 0.002646 | 0.037536474 |
| gmmseq649G000180.1 | *RAI14* | -6634.8 | -6639.29 | 0.002726 | 0.038429886 |
| gmmseq492G000020.1 | *ENTK* | -7168.64 | -7173.09 | 0.002834 | 0.039716434 |
| gmmseq229G000820.1 | *-* | -933.487 | -937.919 | 0.00291 | 0.040524575 |
| gmmseq60G000330.1 | *-* | -3427.5 | -3431.93 | 0.002936 | 0.04064792 |
| gmmseq274G000230.1 | *YF1BB* | -2379.53 | -2383.94 | 0.002973 | 0.040919946 |
| gmmseq245G000380.1 | *SETMR* | -3216.5 | -3220.91 | 0.003004 | 0.041089415 |
| gmmseq89G000400.1 | *-* | -7917.8 | -7922.2 | 0.003006 | 0.040876783 |
| gmmseq750G000210.1 | *CU1A* | -1186.75 | -1191.12 | 0.003111 | 0.042067997 |
| gmmseq138G000100.2 | *DXO* | -1689.48 | -1693.83 | 0.003169 | 0.042593755 |
| gmmseq402G000250.1 | *-* | -1018.62 | -1022.97 | 0.003178 | 0.042472249 |
| gmmseq900G000570.1 | *THG1* | -10294.9 | -10299.2 | 0.003216 | 0.042734237 |
| gmmseq175G000010.1 | *-* | -4858.93 | -4863.24 | 0.003326 | 0.043944 |
| gmmseq115G001380.1 | *RN123* | -1858.88 | -1863.19 | 0.003344 | 0.043929514 |
| gmmseq426G001280.1 | *DDX20* | -8702.37 | -8706.67 | 0.003345 | 0.0436985 |
| gmmseq554G000100.2 | *-* | -3846.19 | -3850.48 | 0.003399 | 0.044149101 |
| gmmseq498G000140.1 | *CCNL1* | -1471.01 | -1475.24 | 0.00366 | 0.047270558 |
| gmmseq110G000670.1 | *IGB1B* | -1754.14 | -1758.35 | 0.003699 | 0.047512498 |
| gmmseq129G002410.1 | *ARMC6* | -3873.36 | -3877.57 | 0.003715 | 0.047455587 |
| gmmseq814G001110.1 | *-* | -1641.42 | -1645.63 | 0.003749 | 0.04762497 |
| gmmseq362G000350.1 | *-* | -2632.08 | -2636.28 | 0.003757 | 0.047464153 |
| gmmseq526G000380.1 | *SUFU* | -2314.53 | -2318.72 | 0.003792 | 0.047642435 |
| gmmseq85G000390.1 | *-* | -2090 | -2094.19 | 0.003798 | 0.047465538 |
| gmmseq373G000110.1 | *SMYD4* | -4177.16 | -4181.32 | 0.003949 | 0.04908131 |
| gmmseq509G000390.1 | *RESIL* | -2822.21 | -2826.36 | 0.003959 | 0.048952727 |
| gmmseq229G000880.1 | *GLCM* | -4403.12 | -4407.26 | 0.004027 | 0.04952659 |

Table S3 The significant enriched pathways based on the identified specifically expressed gene in each tissue in *Morinoia aosen*.

| **GO items enriched in Antennae** | **ID** | **Generatio** | **Corrected P-Value** |
| --- | --- | --- | --- |
| extracellular space | GO:0005615 | 0.011029 | 0.002994 |
| innate immune response | GO:0045087 | 0.053571 | 0.002981 |
| proteolysis | GO:0006508 | 0.008242 | 0.035866 |
| endopeptidase inhibitor activity | GO:0004866 | 0.333333 | 0.002981 |
| positive regulation of Wnt protein secretion | GO:0061357 | 0.285714 | 0.002981 |
| extracellular exosome | GO:0070062 | 0.142857 | 0.00512 |
| microtubule-based process | GO:0007017 | 0.095238 | 0.00894 |
| extracellular matrix organization | GO:0030198 | 0.08 | 0.009973 |
| structural constituent of cytoskeleton | GO:0005200 | 0.076923 | 0.009973 |
| signaling receptor activity | GO:0038023 | 0.051282 | 0.016315 |
| cilium | GO:0005929 | 0.051282 | 0.016315 |
| positive regulation of canonical Wnt signaling pathway | GO:0090263 | 0.05 | 0.016315 |
| microtubule | GO:0005874 | 0.046512 | 0.01715 |
| ligand-gated ion channel activity | GO:0015276 | 0.036364 | 0.024199 |
| endosome | GO:0005768 | 0.033898 | 0.024199 |
| microtubule cytoskeleton organization | GO:0000226 | 0.033333 | 0.024199 |
| metalloendopeptidase activity | GO:0004222 | 0.033333 | 0.024199 |
| cilium assembly | GO:0060271 | 0.030303 | 0.02729 |
| defense response to Gram-negative bacterium | GO:0050829 | 0.022472 | 0.034174 |
| GTPase activity | GO:0003924 | 0.017241 | 0.035866 |
| extracellular matrix | GO:0031012 | 0.017241 | 0.035866 |
| mitotic cell cycle | GO:0000278 | 0.017094 | 0.035866 |
| GTP binding | GO:0005525 | 0.016129 | 0.035866 |
|  |  |  |  |
| **GO items enriched in Compound eye** | **ID** | **Generatio** | **Corrected P-Value** |
| integral component of plasma membrane | GO:0005887 | 0.121711 | 3.51E-25 |
| rhabdomere | GO:0016028 | 0.444444 | 4.98E-13 |
| homophilic cell adhesion via plasma membrane adhesion molecules | GO:0007156 | 0.333333 | 5.48E-12 |
| neuron projection | GO:0043005 | 0.205882 | 8.42E-12 |
| plasma membrane | GO:0005886 | 0.048589 | 7.04E-11 |
| synapse | GO:0045202 | 0.16 | 4.90E-09 |
| chemical synaptic transmission | GO:0007268 | 0.157895 | 4.90E-09 |
| axon extension involved in axon guidance | GO:0048846 | 1 | 4.90E-08 |
| cell-cell adhesion mediated by cadherin | GO:0044331 | 0.666667 | 2.30E-07 |
| identical protein binding | GO:0042802 | 0.333333 | 3.93E-07 |
| synapse organization | GO:0050808 | 0.173077 | 3.93E-07 |
| deactivation of rhodopsin mediated signaling | GO:0016059 | 0.5 | 5.90E-07 |
| calcium ion binding | GO:0005509 | 0.082803 | 5.90E-07 |
| calcium-dependent cell-cell adhesion via plasma membrane cell adhesion molecules | GO:0016339 | 0.461538 | 7.33E-07 |
| phototransduction | GO:0007602 | 0.2 | 7.33E-07 |
| G protein-coupled receptor binding | GO:0001664 | 0.241379 | 1.75E-06 |
| regulation of membrane potential | GO:0042391 | 0.233333 | 2.02E-06 |
| cell-cell adhesion mediator activity | GO:0098632 | 0.555556 | 4.77E-06 |
| neurotransmitter receptor activity | GO:0030594 | 0.285714 | 5.54E-06 |
| neuron projection membrane | GO:0032589 | 0.285714 | 5.54E-06 |
| cadherin binding | GO:0045296 | 0.5 | 6.08E-06 |
| R7 cell development | GO:0045467 | 0.416667 | 1.13E-05 |
| dendrite self-avoidance | GO:0070593 | 0.384615 | 1.48E-05 |
| synaptic vesicle endocytosis | GO:0048488 | 0.214286 | 1.90E-05 |
| neuropeptide signaling pathway | GO:0007218 | 0.148936 | 1.97E-05 |
| nervous system process | GO:0050877 | 0.333333 | 2.35E-05 |
| sensory perception of sound | GO:0007605 | 0.142857 | 2.35E-05 |
| thermotaxis | GO:0043052 | 0.3125 | 2.84E-05 |
| ion transmembrane transport | GO:0034220 | 0.294118 | 3.53E-05 |
| integral component of membrane | GO:0016021 | 0.030263 | 5.28E-05 |
| signaling receptor activity | GO:0038023 | 0.153846 | 8.22E-05 |
| ommatidial rotation | GO:0016318 | 0.208333 | 0.000129 |
| calcium-mediated signaling | GO:0019722 | 0.208333 | 0.000129 |
| heterophilic cell-cell adhesion via plasma membrane cell adhesion molecules | GO:0007157 | 0.208333 | 0.000129 |
| visual perception | GO:0007601 | 0.363636 | 0.000165 |
| enzyme regulator activity | GO:0030234 | 0.192308 | 0.000172 |
| neuropeptide hormone activity | GO:0005184 | 0.185185 | 0.000197 |
| G protein-coupled receptor signaling pathway | GO:0007186 | 0.076923 | 0.000206 |
| axon guidance | GO:0007411 | 0.065217 | 0.000206 |
| rhabdomere development | GO:0042052 | 0.178571 | 0.000213 |
| locomotor rhythm | GO:0045475 | 0.117647 | 0.000253 |
| photoreceptor cell maintenance | GO:0045494 | 0.266667 | 0.000381 |
| signal transduction | GO:0007165 | 0.082353 | 0.000419 |
| axon | GO:0030424 | 0.066116 | 0.000499 |
| motor neuron axon guidance | GO:0008045 | 0.1 | 0.000535 |
| neuron cell-cell adhesion | GO:0007158 | 0.6 | 0.000535 |
| circadian sleep/wake cycle | GO:0042745 | 0.6 | 0.000535 |
| aminergic neurotransmitter loading into synaptic vesicle | GO:0015842 | 0.6 | 0.000535 |
| presynaptic membrane assembly | GO:0097105 | 0.5 | 0.00075 |
| G protein-coupled photoreceptor activity | GO:0008020 | 0.5 | 0.00075 |
| striated muscle thin filament | GO:0005865 | 0.5 | 0.00075 |
| acetylcholine-gated channel complex | GO:0005892 | 0.428571 | 0.001025 |
| acetylcholine-gated cation-selective channel activity | GO:0022848 | 0.428571 | 0.001025 |
| hormone activity | GO:0005179 | 0.173913 | 0.001266 |
| inaD signaling complex | GO:0016027 | 0.375 | 0.001325 |
| neurexin family protein binding | GO:0042043 | 0.375 | 0.001325 |
| cation transport | GO:0006812 | 0.148148 | 0.002087 |
| sensory perception of smell | GO:0007608 | 0.096154 | 0.00215 |
| ion transport | GO:0006811 | 0.3 | 0.00215 |
| ionotropic glutamate receptor complex | GO:0008328 | 0.272727 | 0.002629 |
| phototransduction, visible light | GO:0007603 | 0.272727 | 0.002629 |
| postsynaptic membrane | GO:0045211 | 0.129032 | 0.003099 |
| dendrite | GO:0030425 | 0.065217 | 0.00344 |
| long-term memory | GO:0007616 | 0.083333 | 0.003617 |
| regulation of axonogenesis | GO:0050770 | 0.230769 | 0.003689 |
| cellular response to light stimulus | GO:0071482 | 0.230769 | 0.003689 |
| axonal fasciculation | GO:0007413 | 0.214286 | 0.004381 |
| synaptic vesicle transport | GO:0048489 | 0.1875 | 0.006064 |
| regulation of dendrite morphogenesis | GO:0048814 | 0.166667 | 0.008085 |
| modulation of chemical synaptic transmission | GO:0050804 | 0.157895 | 0.009115 |
| cell surface | GO:0009986 | 0.090909 | 0.009115 |
| extracellular space | GO:0005615 | 0.025735 | 0.009652 |
| sensory perception of chemical stimulus | GO:0007606 | 0.0625 | 0.010758 |
| early endosome | GO:0005769 | 0.085106 | 0.010943 |
| cell-cell junction | GO:0005911 | 0.142857 | 0.010943 |
| cell-cell adhesion | GO:0098609 | 0.142857 | 0.010943 |
| photoreceptor cell axon guidance | GO:0072499 | 0.136364 | 0.011674 |
| protein binding | GO:0005515 | 0.023088 | 0.011674 |
| foregut morphogenesis | GO:0007440 | 0.4 | 0.011674 |
| Wnt-activated receptor activity | GO:0042813 | 0.4 | 0.011674 |
| nephrocyte filtration | GO:0097206 | 0.4 | 0.011674 |
| body fluid secretion | GO:0007589 | 0.4 | 0.011674 |
| store-operated calcium channel activity | GO:0015279 | 0.4 | 0.011674 |
| rhabdomere microvillus membrane | GO:0035997 | 0.4 | 0.011674 |
| regulation of compound eye pigmentation | GO:0048076 | 0.4 | 0.011674 |
| transmembrane signaling receptor activity | GO:0004888 | 0.130435 | 0.011986 |
| adenylate cyclase-activating G protein-coupled receptor signaling pathway | GO:0007189 | 0.130435 | 0.011986 |
| presynaptic active zone | GO:0048786 | 0.130435 | 0.011986 |
| locomotion | GO:0040011 | 0.12 | 0.0143 |
| potassium ion transmembrane transport | GO:0071805 | 0.12 | 0.0143 |
| rhodopsin mediated signaling pathway | GO:0016056 | 0.333333 | 0.0143 |
| regulation of cytosolic calcium ion concentration | GO:0051480 | 0.333333 | 0.0143 |
| compound eye morphogenesis | GO:0001745 | 0.054348 | 0.015127 |
| muscle cell cellular homeostasis | GO:0046716 | 0.115385 | 0.01544 |
| negative regulation of transcription by RNA polymerase II | GO:0000122 | 0.037634 | 0.016333 |
| dipeptidase activity | GO:0016805 | 0.285714 | 0.016333 |
| synaptic signaling | GO:0099536 | 0.285714 | 0.016333 |
| detection of light stimulus involved in visual perception | GO:0050908 | 0.285714 | 0.016333 |
| regulation of myoblast fusion | GO:1901739 | 0.285714 | 0.016333 |
| coreceptor activity | GO:0015026 | 0.285714 | 0.016333 |
| extracellular ligand-gated ion channel activity | GO:0005230 | 0.285714 | 0.016333 |
| synaptic transmission, cholinergic | GO:0007271 | 0.285714 | 0.016333 |
| microtubule cytoskeleton organization | GO:0000226 | 0.066667 | 0.018049 |
| protein homodimerization activity | GO:0042803 | 0.049505 | 0.019177 |
| sensory perception of touch | GO:0050975 | 0.25 | 0.019177 |
| cytoskeleton of presynaptic active zone | GO:0048788 | 0.25 | 0.019177 |
| response to anesthetic | GO:0072347 | 0.25 | 0.019177 |
| trunk segmentation | GO:0035290 | 0.25 | 0.019177 |
| signaling receptor binding | GO:0005102 | 0.096774 | 0.020869 |
| potassium channel activity | GO:0005267 | 0.222222 | 0.021893 |
| entrainment of circadian clock by photoperiod | GO:0043153 | 0.222222 | 0.021893 |
| cGMP biosynthetic process | GO:0006182 | 0.222222 | 0.021893 |
| cGMP-mediated signaling | GO:0019934 | 0.222222 | 0.021893 |
| axon target recognition | GO:0007412 | 0.222222 | 0.021893 |
| instar larval or pupal development | GO:0002165 | 0.222222 | 0.021893 |
| cell elongation involved in imaginal disc-derived wing morphogenesis | GO:0090254 | 0.2 | 0.024828 |
| carbohydrate homeostasis | GO:0033500 | 0.2 | 0.024828 |
| positive regulation of cell migration | GO:0030335 | 0.2 | 0.024828 |
| potassium ion leak channel activity | GO:0022841 | 0.2 | 0.024828 |
| guanylate cyclase activity | GO:0004383 | 0.2 | 0.024828 |
| synaptic target inhibition | GO:0016201 | 0.2 | 0.024828 |
| male courtship behavior | GO:0008049 | 0.083333 | 0.027516 |
| retinal ganglion cell axon guidance | GO:0031290 | 0.181818 | 0.028012 |
| cation channel complex | GO:0034703 | 0.181818 | 0.028012 |
| neuron projection morphogenesis | GO:0048812 | 0.181818 | 0.028012 |
| voltage-gated potassium channel activity | GO:0005249 | 0.181818 | 0.028012 |
| cytoplasmic vesicle | GO:0031410 | 0.078947 | 0.030175 |
| circadian rhythm | GO:0007623 | 0.078947 | 0.030175 |
| membrane | GO:0016020 | 0.028571 | 0.031015 |
| stabilization of membrane potential | GO:0030322 | 0.166667 | 0.031015 |
| endocytic vesicle | GO:0030139 | 0.166667 | 0.031015 |
| cell adhesion molecule binding | GO:0050839 | 0.166667 | 0.031015 |
| cell adhesion | GO:0007155 | 0.076923 | 0.031067 |
| heparin binding | GO:0008201 | 0.153846 | 0.035031 |
| eye development | GO:0001654 | 0.142857 | 0.037829 |
| small GTPase mediated signal transduction | GO:0007264 | 0.142857 | 0.037829 |
| chloride transmembrane transport | GO:1902476 | 0.142857 | 0.037829 |
| carbonate dehydratase activity | GO:0004089 | 0.142857 | 0.037829 |
| positive regulation of circadian sleep/wake cycle, sleep | GO:0045938 | 0.142857 | 0.037829 |
| chloride channel activity | GO:0005254 | 0.142857 | 0.037829 |
| hormone-mediated signaling pathway | GO:0009755 | 0.142857 | 0.037829 |
| peptidase activity | GO:0008233 | 0.133333 | 0.041156 |
| ommochrome biosynthetic process | GO:0006727 | 0.133333 | 0.041156 |
| canonical Wnt signaling pathway | GO:0060070 | 0.133333 | 0.041156 |
| hydro-lyase activity | GO:0016836 | 0.133333 | 0.041156 |
| Wnt-protein binding | GO:0017147 | 0.133333 | 0.041156 |
| galactose binding | GO:0005534 | 0.125 | 0.045087 |
| medium-term memory | GO:0072375 | 0.125 | 0.045087 |
| response to light stimulus | GO:0009416 | 0.125 | 0.045087 |
| potassium ion transport | GO:0006813 | 0.117647 | 0.049736 |
|  |  |  |  |
| **GO items enriched in Gill** | **ID** | **Generatio** | **Corrected P-Value** |
| integral component of membrane | GO:0016021 | 0.011842 | 0.020553 |
| integral component of plasma membrane | GO:0005887 | 0.023026 | 0.003697 |
| plasma membrane | GO:0005886 | 0.010972 | 0.050551 |
| RNA polymerase II transcription regulatory region sequence-specific DNA binding | GO:0000977 | 0.027027 | 0.040237 |
| regulation of intracellular pH | GO:0051453 | 0.6 | 0.000289 |
| open tracheal system development | GO:0007424 | 0.041667 | 0.04192 |
| DNA-binding transcription activator activity, RNA polymerase II-specific | GO:0001228 | 0.034884 | 0.049418 |
| G protein-coupled receptor signaling pathway | GO:0007186 | 0.028846 | 0.053488 |
| DNA-binding transcription factor activity, RNA polymerase II-specific | GO:0000981 | 0.017964 | 0.11219 |
| DNA-binding transcription factor activity | GO:0003700 | 0.014778 | 0.11219 |
| regulation of transcription, DNA-templated | GO:0006355 | 0.012048 | 0.113045 |
| regulation of transcription by RNA polymerase II | GO:0006357 | 0.011236 | 0.113045 |
| sodium:proton antiporter activity | GO:0015385 | 0.4 | 0.012664 |
| photoreceptor cell axon guidance | GO:0072499 | 0.090909 | 0.049418 |
| potassium ion transmembrane transport | GO:0071805 | 0.08 | 0.050551 |
| basolateral plasma membrane | GO:0016323 | 0.076923 | 0.050551 |
| cell surface receptor signaling pathway | GO:0007166 | 0.071429 | 0.053488 |
| neuropeptide receptor activity | GO:0008188 | 0.0625 | 0.053572 |
| positive regulation of border follicle cell migration | GO:1903688 | 0.0625 | 0.053572 |
| salivary gland morphogenesis | GO:0007435 | 0.054054 | 0.066406 |
| wing disc dorsal/ventral pattern formation | GO:0048190 | 0.046512 | 0.083335 |
| proton transmembrane transport | GO:1902600 | 0.039216 | 0.107665 |
| apical plasma membrane | GO:0016324 | 0.038462 | 0.107665 |
| ventral cord development | GO:0007419 | 0.033333 | 0.11219 |
| G protein-coupled receptor activity | GO:0004930 | 0.030769 | 0.11219 |
| structural constituent of chitin-based larval cuticle | GO:0008010 | 0.027778 | 0.11219 |
| structural constituent of cuticle | GO:0042302 | 0.027397 | 0.11219 |
| chitin-based extracellular matrix | GO:0062129 | 0.022222 | 0.11219 |
| compound eye morphogenesis | GO:0001745 | 0.021739 | 0.11219 |
| transcription regulatory region sequence-specific DNA binding | GO:0000976 | 0.021739 | 0.11219 |
|  |  |  |  |
| **GO items enriched in Nerve** | **ID** | **Generatio** | **Corrected P-Value** |
| carbohydrate metabolic process | GO:0005975 | 0.274194 | 1.67E-11 |
| plasma membrane | GO:0005886 | 0.061129 | 1.09E-07 |
| apoptotic process | GO:0006915 | 0.289474 | 1.52E-07 |
| transferase activity, transferring hexosyl groups | GO:0016758 | 0.275 | 2.16E-07 |
| integral component of membrane | GO:0016021 | 0.055263 | 2.97E-07 |
| UDP-glycosyltransferase activity | GO:0008194 | 0.3125 | 3.42E-07 |
| sphingolipid metabolic process | GO:0006665 | 0.636364 | 1.47E-06 |
| proteolysis | GO:0006508 | 0.071429 | 2.45E-06 |
| xenobiotic metabolic process | GO:0006805 | 0.75 | 5.54E-06 |
| scavenger receptor activity | GO:0005044 | 0.5 | 2.63E-05 |
| protein tyrosine kinase activity | GO:0004713 | 0.375 | 8.35E-05 |
| positive regulation of MAPK cascade | GO:0043410 | 0.625 | 8.35E-05 |
| lysosome | GO:0005764 | 0.157895 | 0.000129 |
| CoA-ligase activity | GO:0016405 | 0.333333 | 0.000131 |
| intracellular membrane-bounded organelle | GO:0043231 | 0.104762 | 0.000352 |
| cysteine-type endopeptidase activity involved in execution phase of apoptosis | GO:0097200 | 0.8 | 0.000352 |
| positive regulation of phosphatidylinositol 3-kinase signaling | GO:0014068 | 0.8 | 0.000352 |
| oxidation-reduction process | GO:0055114 | 0.065693 | 0.000352 |
| cytosol | GO:0005829 | 0.04936 | 0.000431 |
| cysteine-type endopeptidase activity involved in apoptotic process | GO:0097153 | 0.666667 | 0.000526 |
| sterol transport | GO:0015918 | 0.666667 | 0.000526 |
| apicolateral plasma membrane | GO:0016327 | 0.294118 | 0.000994 |
| central nervous system development | GO:0007417 | 0.131148 | 0.000994 |
| 3-keto sterol reductase activity | GO:0000253 | 0.5 | 0.001087 |
| transmembrane receptor protein tyrosine kinase activity | GO:0004714 | 0.277778 | 0.001093 |
| positive regulation of kinase activity | GO:0033674 | 0.277778 | 0.001093 |
| positive regulation of ERK1 and ERK2 cascade | GO:0070374 | 0.277778 | 0.001093 |
| long-chain fatty acid metabolic process | GO:0001676 | 0.277778 | 0.001093 |
| neuron cellular homeostasis | GO:0070050 | 0.263158 | 0.00133 |
| long-chain fatty acid-CoA ligase activity | GO:0004467 | 0.25 | 0.001602 |
| determination of adult lifespan | GO:0008340 | 0.080292 | 0.002241 |
| memory | GO:0007613 | 0.227273 | 0.002265 |
| nurse cell apoptotic process | GO:0045476 | 0.363636 | 0.002313 |
| programmed cell death | GO:0012501 | 0.363636 | 0.002313 |
| integral component of plasma membrane | GO:0005887 | 0.055921 | 0.00258 |
| estradiol 17-beta-dehydrogenase activity | GO:0004303 | 0.333333 | 0.002924 |
| amino acid transmembrane transporter activity | GO:0015171 | 0.208333 | 0.002934 |
| multicellular organism development | GO:0007275 | 0.15 | 0.00325 |
| transmembrane transport | GO:0055085 | 0.065657 | 0.00325 |
| receptor complex | GO:0043235 | 0.2 | 0.003286 |
| cellular response to unfolded protein | GO:0034620 | 0.307692 | 0.003447 |
| cell cortex | GO:0005938 | 0.1 | 0.003804 |
| anatomical structure development | GO:0048856 | 0.285714 | 0.004142 |
| transmembrane receptor protein tyrosine kinase signaling pathway | GO:0007169 | 0.185185 | 0.004142 |
| Wnt-protein binding | GO:0017147 | 0.266667 | 0.004867 |
| fatty-acyl-CoA biosynthetic process | GO:0046949 | 0.266667 | 0.004867 |
| metallopeptidase activity | GO:0008237 | 0.25 | 0.005903 |
| oxidoreductase activity | GO:0016491 | 0.090909 | 0.006061 |
| programmed cell death involved in cell development | GO:0010623 | 0.5 | 0.006083 |
| membrane | GO:0016020 | 0.053571 | 0.007079 |
| dipeptidase activity | GO:0016805 | 0.428571 | 0.007889 |
| actin filament-based movement | GO:0030048 | 0.428571 | 0.007889 |
| vacuole | GO:0005773 | 0.428571 | 0.007889 |
| ceramide biosynthetic process | GO:0046513 | 0.428571 | 0.007889 |
| neuron remodeling | GO:0016322 | 0.138889 | 0.011087 |
| triglyceride metabolic process | GO:0006641 | 0.333333 | 0.0135 |
| transmembrane transporter activity | GO:0022857 | 0.08642 | 0.01514 |
| phototransduction | GO:0007602 | 0.125 | 0.016126 |
| dorsal closure | GO:0007391 | 0.084337 | 0.01682 |
| cysteine-type endopeptidase activity | GO:0004197 | 0.166667 | 0.018048 |
| basal part of cell | GO:0045178 | 0.25 | 0.024835 |
| amino acid transmembrane transport | GO:0003333 | 0.142857 | 0.027172 |
| hydrolase activity | GO:0016787 | 0.142857 | 0.027172 |
| chitinase activity | GO:0004568 | 0.230769 | 0.027172 |
| male germ-line stem cell population maintenance | GO:0036098 | 0.230769 | 0.027172 |
| N-glycan processing | GO:0006491 | 0.230769 | 0.027172 |
| contractile ring | GO:0070938 | 0.230769 | 0.027172 |
| cytoplasmic transport, nurse cell to oocyte | GO:0007303 | 0.230769 | 0.027172 |
| extracellular space | GO:0005615 | 0.038603 | 0.027172 |
| heme binding | GO:0020037 | 0.066667 | 0.027532 |
| chitin-based embryonic cuticle biosynthetic process | GO:0008362 | 0.214286 | 0.031236 |
| chitin catabolic process | GO:0006032 | 0.214286 | 0.031236 |
| peptidase activity | GO:0008233 | 0.2 | 0.036206 |
| rhodopsin biosynthetic process | GO:0016063 | 0.2 | 0.036206 |
| calcium ion binding | GO:0005509 | 0.057325 | 0.038401 |
| negative regulation of synaptic growth at neuromuscular junction | GO:0045886 | 0.121212 | 0.04074 |
| regulation of protein localization | GO:0032880 | 0.1875 | 0.04074 |
| metallocarboxypeptidase activity | GO:0004181 | 0.1875 | 0.04074 |
| nervous system development | GO:0007399 | 0.076923 | 0.041876 |
| glucose homeostasis | GO:0042593 | 0.117647 | 0.043688 |
| peptidoglycan binding | GO:0042834 | 0.176471 | 0.045915 |
| immune response | GO:0006955 | 0.114286 | 0.047058 |
| foregut morphogenesis | GO:0007440 | 0.4 | 0.04744 |
| serine-type carboxypeptidase activity | GO:0004185 | 0.4 | 0.04744 |
| long-chain fatty acid biosynthetic process | GO:0042759 | 0.4 | 0.04744 |
| extrinsic component of plasma membrane | GO:0019897 | 0.4 | 0.04744 |
| glycosphingolipid biosynthetic process | GO:0006688 | 0.4 | 0.04744 |
| myosin light chain binding | GO:0032027 | 0.4 | 0.04744 |
| nuclear inner membrane | GO:0005637 | 0.4 | 0.04744 |
| hydrolase activity, hydrolyzing O-glycosyl compounds | GO:0004553 | 0.4 | 0.04744 |
| positive regulation of insulin secretion | GO:0032024 | 0.4 | 0.04744 |
| ecdysis, chitin-based cuticle | GO:0018990 | 0.166667 | 0.04744 |
| regulation of growth | GO:0040008 | 0.166667 | 0.04744 |
|  |  |  |  |
| **GO items enriched in Legs** | **ID** | **Generatio** | **Corrected P-Value** |
| chitin-based cuticle development | GO:0040003 | 0.129032 | 3.27E-14 |
| extracellular matrix | GO:0031012 | 0.112069 | 7.32E-11 |
| chitin-based extracellular matrix | GO:0062129 | 0.111111 | 2.89E-08 |
| structural constituent of chitin-based larval cuticle | GO:0008010 | 0.125 | 6.02E-08 |
| structural constituent of cuticle | GO:0042302 | 0.123288 | 6.02E-08 |
| structural constituent of chitin-based cuticle | GO:0005214 | 0.1875 | 3.64E-06 |
| chitin binding | GO:0008061 | 0.076923 | 1.12E-05 |
| chitin metabolic process | GO:0006030 | 0.085366 | 2.93E-05 |
| oxidation-reduction process | GO:0055114 | 0.036496 | 0.000185 |
| ecdysteroid metabolic process | GO:0045455 | 0.3 | 0.001454 |
| extracellular region | GO:0005576 | 0.02681 | 0.001957 |
| oxidoreductase activity, acting on CH-OH group of donors | GO:0016614 | 0.230769 | 0.002342 |
| sulfotransferase activity | GO:0008146 | 0.25 | 0.031307 |
| oxidoreductase activity | GO:0016491 | 0.045455 | 0.040312 |

Table S4. The 19 hub genes with weight parameter > 0.3 according to the node cytoscape results in talitrids.

| fromNode | toNode | weight | direction |
| --- | --- | --- | --- |
| *VATG* | *RENR* | 0.300615 | undirected |
| *VATS* | *VATA* | 0.30951 | undirected |
| *DACH1* | *VATA* | 0.307511 | undirected |
| *RENR* | *VATA* | 0.307062 | undirected |
| *VATC* | *VATA* | 0.306954 | undirected |
| *ZNT1* | *VATA* | 0.301879 | undirected |
| *VATG* | *VATA* | 0.301239 | undirected |
| *VATF* | *VATA* | 0.300489 | undirected |
| *VATA* | *VATB* | 0.308393 | undirected |
| *RENR* | *VATE* | 0.308833 | undirected |
| *VATC* | *VATE* | 0.306213 | undirected |
| *VATB* | *VATE* | 0.305282 | undirected |
| *ZNT1* | *VATE* | 0.304683 | undirected |
| *VATS* | *VATE* | 0.304055 | undirected |
| *VATF* | *VATE* | 0.301704 | undirected |
| *VATA* | *VATE* | 0.301124 | undirected |
| *VATG* | *VATE* | 0.300765 | undirected |
| *SLC39A1* | *VATE* | 0.300511 | undirected |
| *DACH1* | *VATE* | 0.300191 | undirected |
| *CKAP5* | *VATE* | 0.30006 | undirected |
| *VATB* | *VAT116kDa* | 0.30645 | undirected |
| *VATA* | *VAT116kDa* | 0.305565 | undirected |
| *VATC* | *VAT116kDa* | 0.304904 | undirected |
| *VATS* | *VAT116kDa* | 0.30458 | undirected |
| *VATG* | *VAT116kDa* | 0.304389 | undirected |
| *VATF* | *VAT116kDa* | 0.304161 | undirected |
| *VATE* | *VAT116kDa* | 0.303178 | undirected |
| *RENR* | *VAT116kDa* | 0.301609 | undirected |
| *EHD1* | *VAT116kDa* | 0.301367 | undirected |
| *DACH1* | *VAT116kDa* | 0.30054 | undirected |
| *VATE* | *VATO* | 0.306776 | undirected |
| *VATA* | *VATO* | 0.306294 | undirected |
| *VAT116kDa* | *VATO* | 0.302475 | undirected |
| *VATS* | *VATH* | 0.309528 | undirected |
| *DACH1* | *VATH* | 0.307767 | undirected |
| *VATB* | *VATH* | 0.306949 | undirected |
| *VATA* | *VATH* | 0.306142 | undirected |
| *VAT116kDa* | *VATH* | 0.305132 | undirected |
| *VATO* | *VATH* | 0.304653 | undirected |
| *VATC* | *VATH* | 0.304609 | undirected |
| *RENR* | *VATH* | 0.302689 | undirected |
| *EHD1* | *VATH* | 0.30121 | undirected |
| *VATH* | *GBRB3* | 0.308394 | undirected |
| *VATA* | *GBRB3* | 0.305234 | undirected |
| *VATH* | *KAD1* | 0.314422 | undirected |
| *VATA* | *KAD1* | 0.313569 | undirected |
| *VAT116kDa* | *KAD1* | 0.307497 | undirected |
| *VATE* | *KAD1* | 0.306253 | undirected |
| *VATB* | *KAD1* | 0.305175 | undirected |
| *VATG* | *KAD1* | 0.302514 | undirected |
| *KAD1* | *VATD* | 0.304618 | undirected |
| *RENR* | *VATD* | 0.304596 | undirected |
| *VAT116kDa* | *VATD* | 0.303726 | undirected |
| *VATC* | *VATD* | 0.302805 | undirected |
| *VATB* | *VATD* | 0.302479 | undirected |
| *VATO* | *VATD* | 0.302381 | undirected |
| *VATE* | *VATD* | 0.301526 | undirected |
| *VATA* | *VATD* | 0.301384 | undirected |
| *ZNT1* | *VATD* | 0.300737 | undirected |
| *VATF* | *VATD* | 0.300018 | undirected |
| *VATA* | *VATD* | 0.306261 | undirected |
| *VATH* | *VATD* | 0.300801 | undirected |
| *VATE* | *VATD* | 0.300722 | undirected |
| *BALAT* | *VATD* | 0.300249 | undirected |

Table S5 The significant enriched KEGG pathways based on upregulated genes in *Platorchestia pacifica* gills.

| KEGG Terms | ID | Gene Ratio | P-Value |
| --- | --- | --- | --- |
| Valine, leucine and isoleucine degradation | dme00280 | 0.09091 | 0.001021 |
| Histidine metabolism | dme00340 | 0.25000 | 0.001319 |
| ECM-receptor interaction | dme04512 | 0.16667 | 0.00263 |
| Metabolic pathways | dme01100 | 0.01170 | 0.008792 |
| beta-Alanine metabolism | dme00410 | 0.07692 | 0.010388 |
| Glycine, serine and threonine metabolism | dme00260 | 0.07143 | 0.011869 |
| Pyrimidine metabolism | dme00240 | 0.05000 | 0.02251 |
| Arginine and proline metabolism | dme00330 | 0.03774 | 0.037076 |
| Hippo signaling pathway - fly | dme04391 | 0.03279 | 0.047404 |

Table S6. The DEGs in the gills relative to other tissues and between *M. aosen* and *P. pacifica*. The full name of group could be found in Fig. 4A in the main text.

| GeneID | Gene_name | Group | Foldchange  Gill_others | Foldchange  AG_PPG |
| --- | --- | --- | --- | --- |
| gmmseq306G000130.1 | *KKCC* | PPG_down | -3.784420461 | 2.820049421 |
| gmmseq129G001110.1 | *NRARP* | PPG_down | -3.00587962 | 2.944389282 |
| gmmseq791G000430.1 | *T23O* | PPG_down | -3.312077891 | 3.741612165 |
| gmmseq134G000420.1 | *SLC22A5* | PPG_down | -6.498803098 | 4.997466002 |
| gmmseq206G002270.1 | *TBD2B* | PPG_down | -1.665881621 | 1.094004523 |
| gmmseq485G000610.1 | *RB87F* | PPG_down | -1.534089249 | 1.190359924 |
| gmmseq85G000360.1 | *NXN* | PPG_down | -6.812560409 | 7.154812835 |
| gmmseq206G000980.1 | *-* | PPG_down | -2.235313301 | 2.740879049 |
| gmmseq483G000190.1 | *PLD2* | PPG_down | -1.917373939 | 1.456935793 |
| gmmseq438G000010.1 | *DX39B* | PPG_down | -1.893835187 | 1.21924475 |
| gmmseq137G000800.1 | *DXO* | PPG_down | -3.859614937 | 3.250572731 |
| gmmseq386G000460.1 | *OTUL* | PPG_down | -4.539973674 | 1.824343954 |
| gmmseq428G000200.1 | *-* | PPG_down | -4.648774899 | 3.191472089 |
| gmmseq129G000870.1 | *HAOX1* | PPG_down | -5.495585736 | 3.809940258 |
| gmmseq394G000080.1 | *CBS* | AOSG_down | -2.730300612 | -1.564970001 |
| gmmseq467G001410.1 | *PAHX* | AOSG_down | -2.473838508 | -1.313398104 |
| gmmseq256G001060.1 | *SLC6A13* | AOSG_down | -2.67579867 | -3.931812099 |
| gmmseq81G000300.1 | *RFT2* | AOSG_down | -4.708331973 | -4.103634745 |
| gmmseq771G000070.1 | *TECR* | AOSG_down | -1.603479113 | -1.235280903 |
| gmmseq476G002150.1 | *CO6A6* | AOSG_down | -3.817166467 | -2.998045357 |
| gmmseq591G000480.1 | *SLC36A4* | AOSG_down | -4.709167364 | -3.317597927 |
| gmmseq112G000660.1 | *RGN* | AOSG_down | -4.761240611 | -3.378223751 |
| gmmseq89G000440.1 | *F169B* | AOSG_down | -1.598308655 | -1.018233344 |
| gmmseq164G000260.1 | *-* | AOSG_down | -3.448504932 | -2.474086719 |
| gmmseq142G000570.1 | *ABCB1* | AOSG_down | -6.553191611 | -3.323342918 |
| gmmseq79G000170.1 | *PLPHP* | AOSG_down | -2.054153178 | -1.733495375 |
| gmmseq476G002010.1 | *DL* | AOSG_down | -4.037843848 | -2.152103554 |
| gmmseq59G000740.1 | *UGT2* | AOSG_down | -7.49981684 | -5.449713337 |
| gmmseq260G000090.1 | *KCNK1* | AOSG_down | -5.872590455 | -7.741590033 |
| gmmseq94G000140.1 | *MEX3B* | AOSG_down | -2.83777863 | -2.880644872 |
| gmmseq115G000790.1 | *FAH* | AOSG_down | -2.510979937 | -1.264767849 |
| gmmseq115G000380.2 | *CP2L1* | AOSG_down | -1.899609091 | -1.174818016 |
| gmmseq421G000020.1 | *-* | PPG_up | 4.856803432 | -11.27213999 |
| gmmseq132G001620.1 | *GUTR1* | PPG_up | 2.988037077 | -1.682033693 |
| gmmseq112G000210.1 | *-* | PPG_up | 4.195339976 | -3.910362266 |
| gmmseq259G000060.1 | *CTCF* | PPG_up | 4.6422011 | -4.722060243 |
| gmmseq829G000260.1 | *SCRB2* | PPG_up | 2.49717976 | -1.091798362 |
| gmmseq368G000030.1 | *TINC* | PPG_up | 3.076295016 | -1.962079883 |
| gmmseq129G001950.1 | *-* | PPG_up | 2.73084903 | -1.235984355 |
| gmmseq593G000530.1 | *PHC2* | PPG_up | 2.969928809 | -1.289371992 |
| gmmseq388G000680.1 | *UMPS* | PPG_up | 1.568077712 | -1.037230583 |
| gmmseq1367G000060.1 | *PCCB* | PPG_up | 2.043933394 | -13.79244132 |
| gmmseq740G000160.1 | *TTD14* | PPG_up | 3.318731752 | -1.129462382 |
| gmmseq521G000170.1 | *TGMH* | PPG_up | 2.854423638 | -1.414998501 |
| gmmseq117G000590.1 | *UPP* | PPG_up | 1.869181238 | -2.39283423 |
| gmmseq117G000270.1 | *RGS12* | PPG_up | 2.352147241 | -1.225120327 |
| gmmseq206G001420.1 | *-* | PPG_up | 3.736579612 | -2.308387974 |
| gmmseq89G000750.1 | *VAT1L* | PPG_up | 2.580334712 | -2.013580407 |
| gmmseq1555G000290.1 | *CISYC* | PPG_up | 6.689361143 | -6.995618608 |
| gmmseq591G000620.2 | *TTKB* | PPG_up | 2.24926107 | -1.264762367 |
| gmmseq793G000090.1 | *PCCA* | PPG_up | 1.865497769 | -2.658001523 |
| gmmseq551G000060.1 | *SSR2* | PPG_up | 4.573941112 | -3.521385472 |
| gmmseq363G000150.1 | *ARH* | PPG_up | 4.907152873 | -2.260387342 |
| gmmseq1767G001320.1 | *SODF* | PPG_up | 7.796979222 | -8.101605503 |
| gmmseq112G000190.1 | *Chrna7* | PPG_up | 4.236606451 | -1.502211335 |
| gmmseq814G000850.1 | *POPD1* | PPG_up | 5.07670563 | -2.694620006 |
| gmmseq133G000040.1 | *FABD* | PPG_up | 2.711386557 | -2.708306855 |
| gmmseq247G000970.1 | *ODC* | PPG_up | 1.905061116 | -1.516857562 |
| gmmseq1497G000120.1 | *CNDP1* | PPG_up | 6.99415759 | -7.299508976 |
| gmmseq573G000270.1 | *TOLL6* | PPG_up | 2.240507332 | -4.232737893 |
| gmmseq255G001400.1 | *SLC41A1* | PPG_up | 1.671229651 | -1.660696403 |
| gmmseq476G002430.1 | *Slc22a22* | PPG_up | 3.177156302 | -1.277223474 |
| gmmseq379G000600.1 | *PREP* | PPG_up | 1.205910482 | -1.035809915 |
| gmmseq198G000180.1 | *ATL5* | PPG_up | 3.278341934 | -1.219008383 |
| gmmseq36G000760.1 | *-* | PPG_up | 5.757128078 | -7.456913477 |
| gmmseq379G001230.1 | *CIN* | PPG_up | 2.217116904 | -1.57677761 |
| gmmseq307G000340.1 | *NKCL* | PPG_up | 5.676103482 | -3.514979791 |
| gmmseq312G000310.1 | *EGAL1* | PPG_up | 1.748315989 | -1.063656815 |
| gmmseq421G001510.1 | *APKC* | PPG_up | 1.977850193 | -1.120099607 |
| gmmseq606G000410.1 | *STUB* | PPG_up | 4.165911109 | -2.269927709 |
| gmmseq159G000210.1 | *-* | PPG_up | 3.198414732 | -1.480161306 |
| gmmseq480G000660.1 | *SLC34A1* | PPG_up | 3.221969695 | -2.764635272 |
| gmmseq132G002330.1 | *-* | AG_up | 1.613660931 | 1.992676942 |
| gmmseq1G000260.1 | *FUCTC* | AG_up | 3.961634687 | 7.064366358 |
| gmmseq606G000340.1 | *GLUCL* | AG_up | 5.56706769 | 9.67337717 |
| gmmseq630G000910.1 | *-* | AG_up | 4.776263979 | 2.561420922 |
| gmmseq250G000740.1 | *-* | AG_up | 4.088431167 | 2.668307323 |
| gmmseq115G001990.1 | *NPY2R* | AG_up | 5.022833219 | 3.614039839 |
| gmmseq247G000390.1 | *PCKGM* | AG_up | 2.297078056 | 2.480543147 |
| gmmseq376G000300.1 | *CP315* | AG_up | 4.701695299 | 4.160142828 |
| gmmseq582G000210.1 | *CHHB* | AG_up | 3.330582413 | 2.275854588 |
| gmmseq345G000340.1 | *-* | AG_up | 4.699813242 | 2.446208258 |
| gmmseq509G000240.1 | *TRET1* | AG_up | 5.801100373 | 3.980355073 |
| gmmseq206G001570.1 | *-* | AG_up | 1.463427782 | 2.382596724 |
| gmmseq247G000380.1 | *PCKGM* | AG_up | 5.183075208 | 1.877638984 |
| gmmseq247G001280.1 | *PTGS2* | AG_up | 2.531442443 | 1.646874816 |
| gmmseq65G000200.1 | *MMP21* | AG_up | 6.388133521 | 6.528565261 |
| gmmseq68G000760.1 | *4CLL7* | AG_up | 3.360399033 | 2.329070557 |
| gmmseq8G000010.1 | *-* | AG_up | 4.597555756 | 1.598946524 |
| gmmseq115G001550.1 | *SH24B* | AG_up | 2.001672218 | 1.102964548 |
| gmmseq466G000110.1 | *RHOM1* | AG_up | 2.20559232 | 1.320364343 |
| gmmseq379G000080.1 | *DOXA1* | AG_up | 3.05487252 | 2.121180941 |
| gmmseq754G000110.1 | *WEE1* | AG_up | 2.627957011 | 2.890423504 |
| gmmseq247G001290.2 | *PA24A* | AG_up | 2.273082862 | 1.842003724 |
| gmmseq606G000330.1 | *ENTK* | AG_up | 5.969532778 | 8.728254179 |
| gmmseq447G000680.1 | *-* | AG_up | 4.647806277 | 2.652559436 |
| gmmseq336G000110.1 | *CYGB2* | AG_up | 3.132886992 | 2.182904336 |
| gmmseq736G000100.1 | *Moe* | AG_up | 1.925537642 | 1.092654407 |
